# Near-Infrared Spectroscopy in the Pathophysiology, Diagnosis, and Exercise-Based Management of Muscle Oxygenation Impairment

**DOI:** 10.3390/diagnostics16111585

**Published:** 2026-05-22

**Authors:** Junyan Liu, Nicolas C. Kelhofer, Tyler S. Burtner, W. Catherine Cheung, Manuel E. Hernandez, Yih-Kuen Jan

**Affiliations:** 1 Department of Health and Kinesiology, University of Illinois Urbana-Champaign, Urbana, IL 61801, USA; 2 Carle Illinois College of Medicine, University of Illinois Urbana-Champaign, Urbana, IL 61801, USA; nck7@illinois.edu (N.C.K.); burtner3@illinois.edu (T.S.B.); 3 Doctor of Physical Therapy Program, Northern Illinois University, DeKalb, IL 60115, USA; wcheung@niu.edu

**Keywords:** exercise training, near-infrared spectroscopy, oxygen transport cascade, peripheral artery disease, rehabilitation, scoping review

## Abstract

Muscle oxygen nation impairment, defined as a mismatch between oxygen delivery, distribution, and oxidative utilization in active skeletal muscle, contributes to exercise intolerance and functional decline. Near-infrared spectroscopy (NIRS) has emerged as the leading non-invasive tool for monitoring local muscle oxygenation, but its clinical translation and optimal exercise-based management remain incompletely defined. This scoping review aimed to (1) synthesize the pathophysiology of muscle oxygenation impairment across the oxygen transport cascade, (2) evaluate NIRS-based diagnostic protocols, and (3) review exercise-based interventions targeting muscle oxygenation. The review followed PRISMA-ScR guidelines and was prospectively registered in OSF (DOI: 10.17605/OSF.IO/QW8R3) and PROSPERO (CRD420261365040). PubMed, Web of Science, Scopus, Cochrane CENTRAL, EMBASE, PEDro, and ClinicalTrials.gov were searched through to April 2026. Methodological quality was appraised using the PEDro scale, the Downs and Black checklist, and the Newcastle–Ottawa Scale. A total of 61 studies (2003–2025) were included, with fair-to-good methodological quality (PEDro 3–8, mean 5.3; Downs and Black 15–24, mean 18.6; Newcastle–Ottawa 5–8, mean 6.5). Regarding pathophysiology, muscle oxygenation impairment is a cascade-level phenomenon with four mechanistically distinct phenotypes corresponding to the dominant site of impairment, each with characteristic NIRS signatures. Regarding diagnostic assessment, NIRS has shown value in selected contexts including a validated threshold for peripheral artery disease, but most studies report group-level correlations without deriving receiver operating characteristic curves at validated thresholds, which together with device and calibration heterogeneity limits clinical translation. Regarding exercise-based interventions, adaptations align with the underlying cascade lesion, sprint and high-intensity interval training enhance oxidative capacity, while walking-based and vascular-targeted programs preferentially improve microvascular function. These findings support a unifying framework in which the site of cascade impairment guides diagnostic protocol selection and exercise prescription. The proposed cascade lesion phenotyping schema is hypothesis-generating and requires prospective validation.

## 1. Introduction

Skeletal muscle is the most metabolically dynamic tissue during physical activity, with oxygen consumption in trained healthy muscle capable of increasing 50- to 100-fold above resting levels during intense exercise [1,2]; achievable ranges are substantially lower in the clinical populations discussed throughout this review. This extraordinary metabolic range is sustained by an integrated oxygen transport system in which atmospheric oxygen moves through a series of convective and diffusive steps, from ventilation and pulmonary gas exchange, through cardiac output and arterial delivery, to microvascular perfusion and, ultimately, diffusion across the capillary wall, interstitium, and sarcolemma to reach mitochondria [2,3]. The structural and functional components of this pathway, including pulmonary diffusing capacity, cardiac chambers, hemoglobin mass, skeletal muscle capillary networks, and mitochondrial density, are closely matched to the maximal oxidative demands of the organism, especially those of skeletal muscle during exercise, such that disruption at any level can compromise the entire pathway [4]. Accordingly, adequate muscle oxygenation, defined as the dynamic balance between oxygen delivery from the microcirculation and oxygen utilization by muscle mitochondria, is essential not only for force production, endurance, and exercise tolerance but also for tissue viability and metabolic homeostasis [1,5,6]. When this balance is disrupted, the consequences extend beyond reduced exercise capacity to include accelerated fatigue, impaired metabolic recovery, and progressive functional decline across a broad spectrum of clinical populations [7,8,9].

Muscle oxygenation impairment, defined as an abnormal mismatch between oxygen delivery to, distribution within, and oxidative utilization by active skeletal muscle, regardless of whether the primary limitation originates centrally, peripherally, or within the muscle itself, is increasingly recognized as a common pathophysiological feature across diverse clinical conditions [6,7]. For the purposes of this review, muscle oxygenation impairment is operationally defined as a deviation from age- and sex-matched normative NIRS responses, manifesting as one or more of the following: reduced peak deoxygenation amplitude or extraction during exercise; prolonged tissue oxygen saturation or deoxygenated hemoglobin recovery time (e.g., T50 > 70 s in PAD); a Δ[HHb]/ΔVO_2_ profile indicative of delivery–utilization mismatch; or a blunted hyperemic response following arterial occlusion. We note that universal device-independent NIRS thresholds remain to be established for most conditions, and the absence of such thresholds is itself a key gap identified by this review. In chronic heart failure (CHF), chronic obstructive pulmonary disease (COPD), peripheral artery disease (PAD), critical illness, congenital heart disease (CHD), and aging-related frailty, disturbances at different levels of the oxygen transport pathway converge to produce exercise intolerance, accelerated fatigue, and functional decline [7,8,10,11,12,13,14]. These disturbances may involve reduced convective delivery, impaired microvascular oxygen distribution, compromised diffusive transport, diminished oxidative utilization, or any combination of these mechanisms [1,15]. Importantly, impairment often becomes most evident during exercise or metabolic stress, highlighting the need for dynamic assessment methods [5,8]. Historically, assessment of muscle oxygenation relied on invasive methods, such as arteriovenous oxygen difference measurement via catheterization, muscle biopsy with histochemical or biochemical analysis of oxidative enzyme activity and capillary density, and laboratory-based phosphorus magnetic resonance spectroscopy (^31^P-MRS) for evaluating mitochondrial function [5,16,17]. Although these approaches have provided foundational insight into muscle oxidative metabolism, they are limited by invasiveness, high cost, lack of portability, and inability to provide continuous real-time monitoring during exercise, underscoring the need for more accessible diagnostic strategies.

In contrast to these traditional approaches, near-infrared spectroscopy (NIRS) has emerged as the most widely used non-invasive tool for monitoring local muscle oxygenation, offering portable, real-time, and continuous assessment of the balance between oxygen delivery and utilization without requiring catheterization or tissue sampling [5,6,18]. Whereas ^31^P-MRS provides complementary insight into mitochondrial oxidative function, it remains confined to specialized laboratory settings and cannot be applied during free-living exercise [16,17]. Cardiopulmonary exercise testing (CPET) remains the gold-standard integrative test of exercise physiology and systemic oxygen transport limitation, although it does not directly resolve local skeletal muscle oxygenation [10], while magnetic resonance spectroscopy offers complementary insight into mitochondrial oxidative function [16,17]. Emerging technologies including wearable multi-channel NIRS, diffuse correlation spectroscopy, and contrast-enhanced ultrasound hold promise for expanding clinical applicability [19,20,21]. However, each modality carries significant limitations, and no single tool currently captures all aspects of muscle oxygenation across the full oxygen transport pathway. In clinical practice, determining whether exercise intolerance in an individual patient results primarily from impaired convective oxygen delivery, compromised microvascular diffusion, or diminished mitochondrial utilization remains challenging, and this diagnostic uncertainty can misdirect therapeutic decisions. Beyond diagnosis, exercise-based interventions are particularly relevant because they may target multiple determinants of muscle oxygenation simultaneously, including vascular function, capillary density, and oxidative capacity [13,22,23,24,25,26], yet the optimal prescription and monitoring strategies remain incompletely defined.

In summary, muscle oxygenation is governed by an integrated cascade of convective and diffusive oxygen transport steps, and impairment at any level of this cascade, including cardiovascular, pulmonary, microvascular, or mitochondrial dysfunction, can produce exercise intolerance and functional decline across diverse clinical populations. Diagnostic capabilities have advanced substantially, from invasive catheterization and biopsy-based approaches to non-invasive real-time monitoring with NIRS and emerging technologies, yet no single tool captures all aspects of muscle oxygenation across the full transport pathway, and diagnostic uncertainty continues to complicate clinical decision-making. Similarly, although exercise-based interventions can target multiple determinants of muscle oxygenation simultaneously [13,22,23,24,25], optimal prescription and monitoring strategies remain incompletely defined. Several existing reviews have advanced understanding of specific aspects of this field. Foundational reviews on NIRS methodology and its application to skeletal muscle have provided detailed accounts of measurement principles, signal interpretation, and technical limitations [5,6,8,18], while physiological reviews have elucidated the mechanisms of oxygen transport from capillary to mitochondria and the dynamic heterogeneity of muscle blood flow and oxygen utilization during exercise [1,2,15]. Disease-specific reviews have examined the pathophysiology of muscle oxygenation impairment within individual conditions, including heart failure [7,10], comorbid COPD and heart failure [11], critical illness [12], and congenital heart disease [14], and population-specific systematic reviews have evaluated exercise training effects on muscle oxygenation in older adults [13]. A recent narrative review by our group [9] examined oxygen-related therapeutic interventions in selected clinical conditions and provided a partial foundation for the present synthesis; however, that work did not apply a systematic PRISMA-ScR search, did not employ formal risk-of-bias appraisal, did not address NIRS as the central diagnostic modality, and did not develop a cascade-level phenotyping framework linking diagnostic protocol selection to exercise prescription. However, these contributions have generally remained focused on either the measurement technology, the underlying physiology, or a single clinical population, without systematically integrating the pathophysiological mechanisms across the full oxygen transport cascade, the comparative strengths and limitations of available diagnostic modalities, and the evidence for exercise-based management within a unified clinical framework. In particular, no existing systematic review has integrated the question of where along the oxygen cascade impairment originates with how diagnostic and therapeutic strategies can be matched accordingly across diverse patient populations within a unified mechanistic framework. A comprehensive framework that integrates the pathophysiology, diagnostic assessment, and management of muscle oxygenation impairment across clinical populations is therefore needed. The objectives of this review are to: (1) synthesize the physiological and pathological mechanisms of muscle oxygenation impairment across the oxygen transport cascade in both health and disease; (2) evaluate NIRS-based diagnostic protocols and the complementary value of other diagnostic modalities for assessing muscle oxygenation in clinical practice; (3) review the evidence for exercise-based and rehabilitative interventions targeting muscle oxygenation; and (4) identify critical gaps and future research directions to advance the clinical translation of NIRS-based muscle oxygenation assessment and management.

## 2. Methods

### 2.1. Study Design

This study was conducted as a systematic scoping review following the PRISMA extension for scoping review (PRISMA-ScR) guidelines [27]. A scoping review design was selected owing to the substantial heterogeneity in study populations, intervention protocols, NIRS instrumentation, outcome measures, and testing paradigms across the included literature, which precluded quantitative pooling. The review protocol was registered prospectively in the Open Science Framework (OSF) [registration DOI: 10.17605/OSF.IO/QW8R3] and in the International Prospective Register of Systematic Reviews (PROSPERO; registration number CRD420261365040). Both registrations were prospective and were not subjected to external peer review prior to study commencement, in line with the standard registration procedures of both platforms.

The decision to use narrative synthesis rather than quantitative meta-analysis was made a priori and documented in the registered protocol. Although two subgroups (sprint and repeated-sprint training in healthy and trained participants, *n* = 10; and walking-based programs in peripheral artery disease, *n* = 8) contained sufficient studies to consider partial quantitative pooling, the persistent within-subgroup heterogeneity in NIRS device type (continuous wave, frequency domain, broadband), outcome metric (e.g., deoxygenated hemoglobin amplitude, kinetic time constants, reoxygenation T50, Δ[HHb]/ΔVO_2_ profile), training protocol (sprint number, work-rest ratio, training duration), and calibration approach (baseline referencing, cuff occlusion, manufacturer) precluded a methodologically defensible pooled estimate. Future reviews focused on a single device family with harmonized outcomes may be in a position to perform partial meta-analysis.

### 2.2. Search Strategy

A systematic literature search was conducted in PubMed, Web of Science, Scopus, the Cochrane Central Register of Controlled Trials (CENTRAL), EMBASE, the Physiotherapy Evidence Database (PEDro), and ClinicalTrials.gov from database inception through to April 2026. The strategy combined three conceptual domains using Boolean operators: (1) muscle oxygenation (e.g., “muscle oxygenation,” “tissue oxygenation,” “SmO_2_,” “StO_2_,” “TSI,” “TOI,” “HHb,” “O_2_Hb,” “tHb”); (2) near-infrared spectroscopy (e.g., “near-infrared spectroscopy,” “NIRS”); and (3) exercise interventions (e.g., “exercise training,” “resistance training,” “HIIT,” “sprint interval training,” “rehabilitation,” “blood flow restriction,” “inspiratory muscle training”). MeSH or platform-equivalent subject headings and free-text keywords were combined within each domain using OR and intersected across domains using AND. The search was adapted for each platform and restricted to English-language publications; for EMBASE, records originating in MEDLINE were excluded to avoid duplication with the PubMed results. Complete database-specific search strategies are provided in Table S1. Reference lists of included studies and relevant reviews were hand-searched to identify additional records.

### 2.3. Eligibility Criteria

Studies were included if they met all of the following PICOS criteria: (1) population—human participants of any age, sex, training status, or health condition, including healthy individuals and clinical populations; (2) intervention—an exercise training intervention, an acute physiological experiment involving exercise, or an observational or cross-sectional assessment characterizing muscle oxygenation in clinical populations; (3) comparator—any or none, as studies with and without comparator groups were eligible; (4) outcome—NIRS-measured skeletal muscle oxygenation evaluated during or following exercise or provocative testing; and (5) study design—full-text peer-reviewed original research published in English. Studies were excluded if they (1) did not measure skeletal muscle oxygenation using NIRS (e.g., cerebral NIRS only, pulse oximetry, or non-NIRS modalities); (2) were conference abstracts, editorials, reviews, case reports, or animal studies; or (3) did not provide sufficient methodological detail to permit meaningful data extraction. For criterion (3), a study was deemed to lack sufficient methodological detail if it failed to report at least three of the following four core items: (a) NIRS device manufacturer and model; (b) target muscle and anatomical landmark for probe placement; (c) exercise or provocative test protocol; and (d) NIRS-derived outcome variable. Case reports and single-subject studies were excluded because group-level data are required to characterize patterns across populations and protocols, which is the central objective of this scoping review; this exclusion is consistent with the published scoping review methodology guidance from the Joanna Briggs Institute and does not preclude clinical relevance of single-subject NIRS observations, which are addressed elsewhere in the literature.

### 2.4. Study Selection

Retrieved records were imported into EndNote and duplicates removed. Two reviewers (TB and NK) independently screened titles and abstracts, then assessed full texts of potentially relevant records against eligibility criteria. Disagreements were resolved through discussion or consultation with the corresponding author (YKJ). The selection process was documented using a PRISMA flow diagram. Inter-reviewer agreement was quantified using Cohen’s kappa coefficient. Agreement was κ = 0.82 (95% CI: [0.77–0.87] for title/abstracts and κ = 0.88 (95% CI: [0.82–0.94]) for full texts.

### 2.5. Data Extraction

Two reviewers (JL and TB) independently extracted data using a standardized extraction form (Appendix B). All studies were independently double extracted; discrepancies were identified field by field and resolved through consensus, with the corresponding author (YKJ) consulted when needed. Across 62 studies independently re-extracted by both reviewers, percent agreement across extracted fields was 91%. The form captured four domains: (1) study identification (authors, year, study design); (2) participant characteristics (sample size, sex, age, training status, health status); (3) intervention details (exercise type, duration, frequency, intensity, progression, and comparator); and (4) NIRS methodology (device and measurement technology, target muscle and probe placement, source–detector spacing, sampling rate, adipose tissue correction, calibration/normalization approach, NIRS-derived outcomes, and test protocol). The complete extraction framework is presented in Table 1.

### 2.6. Quality Assessment

Given the heterogeneity of study designs, multiple appraisal tools were employed: the PEDro scale [28] for randomized controlled trials (scored 0–10), the Downs and Black checklist [29] for non-randomized intervention studies (27 items), and the Newcastle–Ottawa Scale adapted for cross-sectional studies [30] and for observational and cross-sectional designs. Two reviewers (TB and NK) independently assessed each study, with disagreements resolved through discussion or consultation with the corresponding author (YKJ). Quality scores were used descriptively to contextualize the evidence; no studies were excluded based on the quality assessment.

### 2.7. Data Synthesis

Data were synthesized narratively owing to the substantial heterogeneity in populations, interventions, NIRS instrumentation, outcome measures, and testing protocols, which precluded quantitative meta-analysis. Findings from intervention studies were organized by exercise modality and by clinical population. Cross-sectional studies were synthesized to characterize patterns of muscle oxygenation impairment across disease states relative to healthy controls. NIRS methodological characteristics were tabulated descriptively to evaluate current practice and identify areas of inconsistency. For each intervention study, the direction and magnitude of training-induced changes in NIRS-derived outcomes were extracted in the units provided by the original publication (absolute values, percentage change, effect size, or kinetic time constants) without further transformation; when only inferential statistics were available, the direction of change and reported *p*-value were extracted. Summary tables presenting the characteristics and main findings of all included studies are provided.

The decision to use narrative synthesis was made a priori and documented in the registered protocol. Although two subgroups (SIT/RST in healthy participants, n = 10; walking-based programs in PAD, n = 8) contained sufficient studies to consider partial pooling, within-subgroup heterogeneity in NIRS device type, outcome metric, training protocol, and calibration approach precluded a methodologically defensible pooled estimate.

## 3. Results

### 3.1. Study Selection and Characteristics

**Figure 1:**
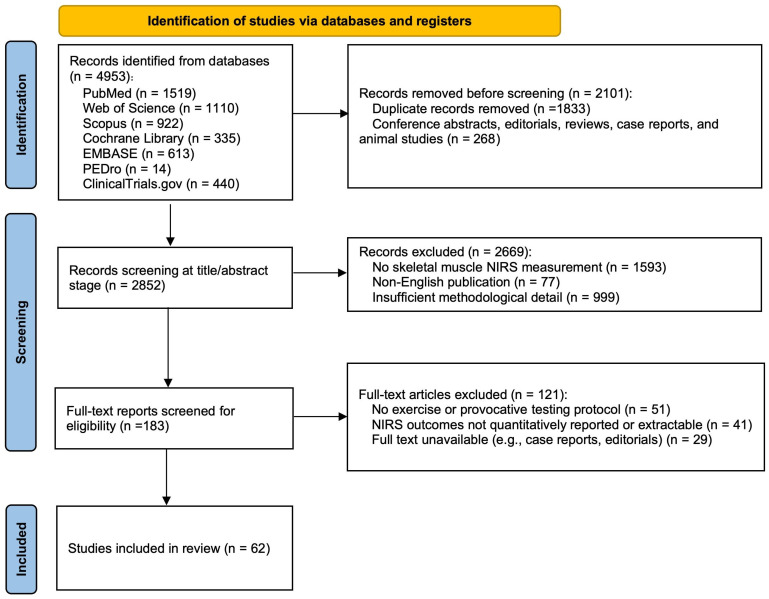
PRISMA flow diagram.

The systematic search yielded 4953 records across seven databases (PubMed 1519; Web of Science 1110; Scopus 922; EMBASE 613; ClinicalTrials.gov 440; Cochrane CENTRAL 335; PEDro 14). After the removal of duplicates and records excluded by document type, records were screened at the title and abstract stage. Following a full-text review, 62 studies were retained for synthesis. The complete selection process is detailed in the PRISMA flow diagram (Figure 1). The included studies were published between 2003 and 2025, encompassing 27 randomized controlled trials [23,25,31,32,33,34,35,36,37,38,39,40,41,42,43,44,45,46,47,48,49,50,51], six non-randomized controlled trials [52,53,54,55,56,57], 17 pre–post intervention studies [24,58,59,60,61,62,63,64,65,66,67,68,69,70,71,72,73], and 10 cross-sectional or observational designs [14,74,75,76,77,78,79,80,81,82]. Two additional study designs (a single multicenter cross-sectional study and a crossover trial) accounted for the remaining records.

Studies were grouped by primary clinical subgroup in Table 1, with internal subheaders providing rapid access to the per-population evidence base. The largest subgroup comprised healthy or trained adults (*n* = 26) [31,33,34,35,36,39,40,41,42,43,44,45,49,50,51,56,62,63,64,65,67,73,83,84,85], followed by chronic obstructive pulmonary disease (*n* = 9) [24,32,37,53,59,71,74,80,81], peripheral artery disease (*n* = 8) [23,46,47,48,52,58,61,86], congenital heart disease (*n* = 3) [38,55,75], type 1 or type 2 diabetes (*n* = 3) [54,72,76], chronic kidney disease or ESRD (*n* = 3) [79,82,87], a single pulmonary arterial hypertension cohort [14], and four studies in other clinical populations (breast cancer, post-ACL reconstruction, metabolic myopathy, and mechanically ventilated ICU patients) [43,51,80,86].

Across the 62 included studies, 1403 participants had reported sex data, of whom 393 (28.0%) were women. The proportion of women varied substantially across subgroups: 20.5% in healthy/trained cohorts, 19.0% in COPD, 32.3% in PAD/intermittent claudication, 26.7% in CHF/HFrEF, 36.9% in CKD/ESRD, 25.0% in diabetes, 33.3% in congenital heart disease, 0% in the post-ACL reconstruction study, 46.2% in metabolic myopathy, and 100% in the single breast cancer trial. Participant ages spanned 12–75 years, with pediatric and adolescent representation limited to congenital heart disease cohorts. Five studies (Moalla 2006, Moalla 2012, Panagiotou 2016, Paradis-Deschênes 2020, Pramkratok 2022) did not report sex breakdown and are excluded from the denominator [55,71,72,75,83].

Among the 49 intervention studies, exercise modalities included sprint and repeated-sprint training (*n* = 10) [31,33,35,39,40,41,42,43,57,83], high-intensity interval training (*n* = 9) [24,34,36,38,49,51,53,63,66], walking-based programs (*n* = 8) [23,46,47,48,52,58,61,86], continuous aerobic training (*n* = 6) [50,56,59,64,67,84], combined or multimodal rehabilitation (*n* = 5) [25,55,60,71,72], resistance training (*n* = 3) [44,45,87], blood flow restriction training (*n* = 5) [25,33,44], inspiratory muscle training (*n* = 2) [70,85], and other modalities (*n* = 3) [54,65,73]. Training durations ranged from two weeks to over six months, with most programs lasting four to twelve weeks. The remaining 12 studies characterized muscle oxygenation patterns across populations without a training intervention [14,68,69,74,75,76,77,78,79,80,81,82]. The vastus lateralis was the most commonly measured muscle (*n* = 36), followed by the gastrocnemius or calf complex (*n* = 12), upper-limb and forearm muscles (*n* = 6), respiratory muscles (*n* = 3), and other sites including the rectus femoris, quadriceps, and tibialis anterior (*n* = 3). Full characteristics of the included studies are presented in Table 1.

### 3.2. Quality Assessment

**Figure 2:**
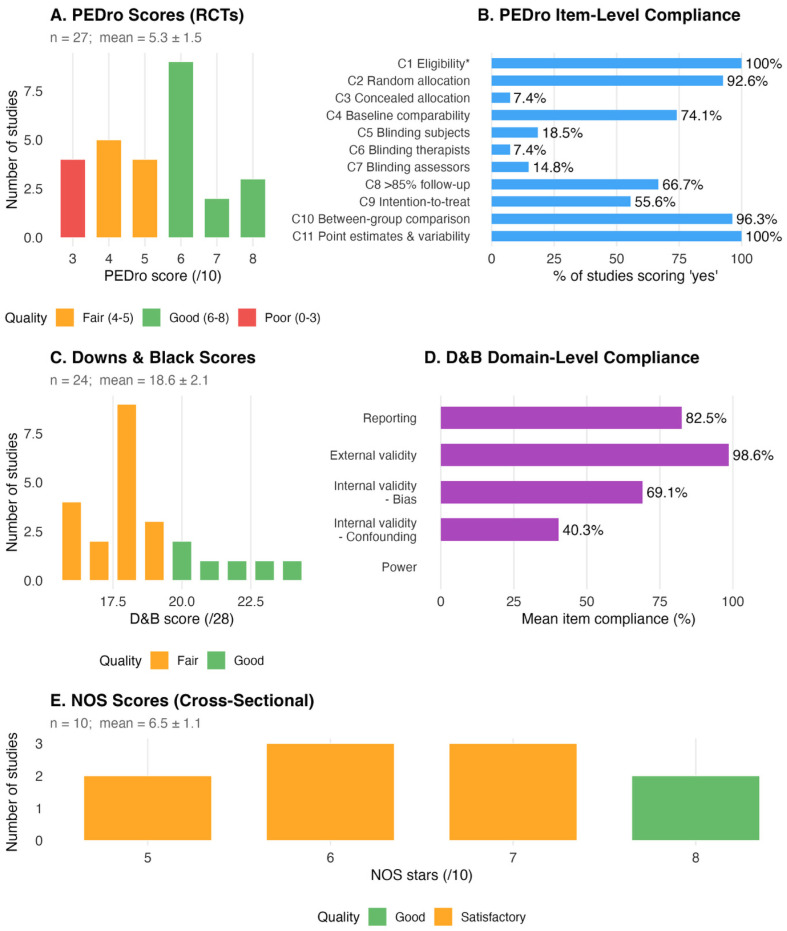
Quality assessment of included studies. (**A**) Distribution of PEDro scores for randomized controlled trials; (**B**) item-level PEDro compliance (percentage of RCTs scoring “yes” on each item); (**C**) distribution of Downs & Black scores; (**D**) Downs & Black domain-level mean item compliance; (**E**) distribution of Newcastle–Ottawa Scale (NOS) scores for cross-sectional studies. * PEDro item 1 (eligibility criteria) relates to external validity and is not included in the total PEDro score (0–10).

Among the 27 RCTs, PEDro scores ranged from 3 to 8 (mean 5.3 ± 1.5), with most studies rated as fair to good quality (Figure 2A). Nearly all RCTs reported random allocation (92.6%), between-group comparisons (96.3%), and point estimates with variability (100%), but concealed allocation (7.4%) and blinding of participants (18.5%), therapists (7.4%), and assessors (14.8%) were rarely achieved (Figure 2B), consistent with the inherent difficulty of blinding exercise interventions. The 24 non-randomized intervention studies scored 15–24 on the Downs and Black checklist (mean 18.6 ± 2.1), with strong reporting (82.5%) and external validity (98.6%) but weak confounding adjustment (40.3%) and absent power analyses (Figure 2C,D). The 10 cross-sectional studies scored 5–8 on the Newcastle–Ottawa Scale (mean 6.5 ± 1.1) and rated as satisfactory to good (Figure 2E). No studies were excluded based on quality. Quality scores for individual studies are reported in Supplementary Tables S1–S3.

While participant and therapist blinding is inherently difficult in exercise trials, the low rate of assessor blinding for NIRS-derived outcomes (14.8%) is methodologically consequential. Because NIRS signal acquisition involves probe placement, signal quality checks, and analytic windowing decisions, all of which can be influenced by knowledge of group allocation, the absence of assessor blinding constrains the certainty with which observed training effects can be causally attributed to the intervention.

### 3.3. NIRS Methodology

**Figure 3:**
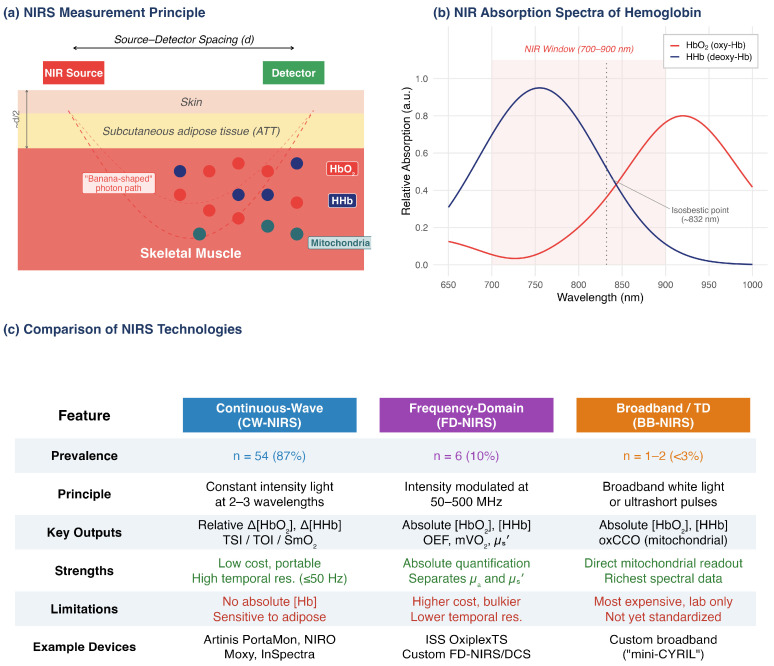
Near-infrared spectroscopy (NIRS): measurement principles and device comparison. (**a**) Schematic of the NIRS measurement principle, showing source–detector geometry and the “banana-shaped” photon path through skin, subcutaneous adipose tissue, and skeletal muscle. (**b**) NIR absorption spectra of oxygenated (HbO_2_) and deoxygenated (HHb) hemoglobin across the NIR window (700–900 nm), with the isosbestic point (~832 nm). (**c**) Comparison of continuous-wave (CW-NIRS), frequency-domain (FD-NIRS), and broadband/time-domain (BB-NIRS) technologies. In panel (**c**), green text denotes relative strengths and red text denotes limitations; colored prevalence values are matched to their respective technology columns.

The underlying measurement principle, comparative features of the three main NIRS technology types, and their relative adoption across the included studies are summarized in Figure 3. The 61 included studies employed a wide range of NIRS instrumentation, measurement protocols, and analytical approaches (Table 2). The majority used continuous-wave (CW) devices (*n* = 54), while six used frequency-domain (FD) systems [25,46,47,52,60,70], one used a hybrid FD-NIRS and diffuse correlation spectroscopy platform [23], and one used a broadband system capable of measuring cytochrome c oxidase oxidation state [45]. Devices from Hamamatsu (*n* = 18) and Artinis (*n* = 17) were most frequent, followed by ISS (*n* = 5), Hutchinson InSpectra (*n* = 5), NIM/NIMS (*n* = 5), ASTEM (*n* = 3), OmegaWave (*n* = 2), and Moxy (*n* = 2). The strong predominance of CW systems (*n* = 54, 88.5%) over FD (*n* = 6, 9.8%) and broadband devices (*n* = 1, 1.6%) constrains the body of evidence to relative measurements such as percentage change, kinetic time constants, and normalized amplitudes; absolute quantification of oxygen extraction fraction and muscle metabolic rate of oxygen, which requires FD or time-resolved devices, was possible in only a small minority of studies. This methodological asymmetry limits direct cross-study comparison of absolute oxygenation values and is revisited as a cross-cutting limitation in Section 4.5.

Substantial variability was observed in source–detector configurations, calibration procedures, and NIRS-derived outcome reporting across the included studies. Source–detector spacing ranged from 12.5 to 50 mm, and sampling rates from 0.33 to 50 Hz. Calibration and normalization procedures varied substantially. Baseline referencing was the most common approach (*n* = 43), followed by physiological calibration using cuff occlusion to define a 0–100% deoxygenation scale (*n* = 13) and manufacturer or device-specific calibration (*n* = 6). Quantitatively, source–detector spacing was reported in 63.9% of studies (39 of 61), sampling rate in 67.2% (41 of 61), and adipose tissue thickness in only 37.7% (23 of 61); reporting rates for the full set of key methodological parameters are presented in Supplementary Figure S1, ranging from 100% (device manufacturer, target muscle) to 11.5% (differential pathlength factor). The combined gap in source–detector spacing, sampling rate, and adipose tissue correction is especially consequential because these parameters jointly determine the effective penetration depth, signal-to-noise ratio, and quantitative validity of the NIRS measurement, and the absence of standardized reporting precludes meaningful between-study comparison, even within a single device family. Outcome variable reporting was similarly heterogeneous; deoxygenated hemoglobin (HHb) was the most widely used variable, serving as a surrogate for fractional oxygen extraction, while tissue oxygen saturation was reported under multiple designations (StO_2_, TSI, TOI, SmO_2_, StiO_2_), reflecting device-specific nomenclature rather than distinct physiological constructs.

Several studies derived kinetic parameters (τ, TD, MRT, reoxygenation rates) from exponential curve fitting, while more advanced approaches included area under the curve calculations [42,48,63], spatial heterogeneity indices from multi-channel systems [64,73,74], wavelet-based spectral analysis [82], and entropy-based non-linear signal analysis [69]. In summary, the predominance of CW technology, lack of standardized calibration protocols, inconsistent adipose tissue correction, high rates of incomplete methodological reporting, and multiple nomenclatures for functionally similar indices limit direct cross-study comparison and provide direct empirical support for the minimum reporting standards proposed.

### 3.4. Pathophysiology of Muscle Oxygenation Impairment Across Clinical Populations

**Figure 4:**
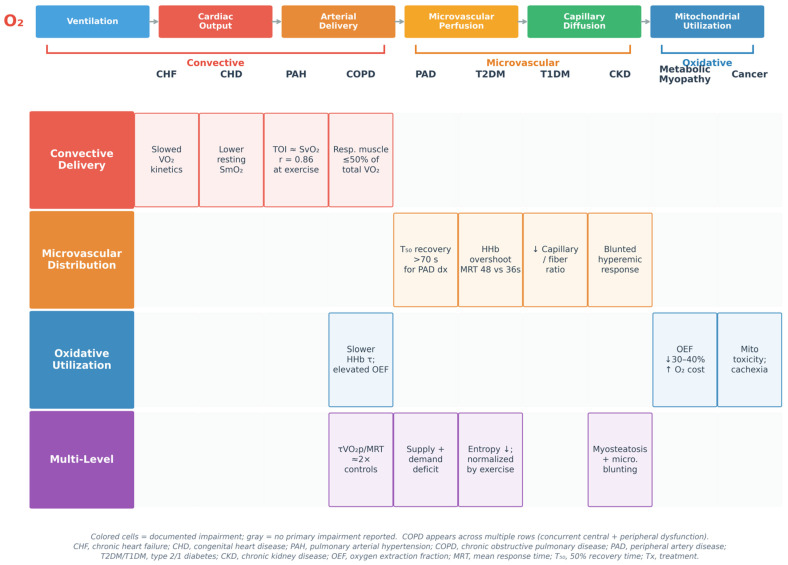
Pathophysiology of muscle oxygenation impairment across the oxygen transport cascade. The top band depicts the six sequential steps of the oxygen cascade; gray arrows indicate the direction of oxygen transport, and the colored brackets group the steps into convective, microvascular, and oxidative phases. The matrix maps documented impairment phenotypes (rows) to clinical populations (columns): colored cells indicate a documented impairment at that level, and gray cells indicate no primary impairment reported for that combination. Within cells, ↑/↓ denote a reported increase/decrease and ≈ denotes an approximate relationship. CHF, chronic heart failure; CHD, congenital heart disease; PAH, pulmonary arterial hypertension; COPD, chronic obstructive pulmonary disease; PAD, peripheral artery disease; T2DM/T1DM, type 2/1 diabetes; CKD, chronic kidney disease; OEF, oxygen extraction fraction; MRT, mean response time; T_50_, 50% recovery time; Tx, treatment.

The pathophysiological mechanisms of muscle oxygenation impairment identified across the included studies are organized along the oxygen transport cascade and mapped to clinical populations in Figure 4, encompassing impaired convective oxygen delivery, impaired microvascular distribution and diffusive transport, impaired oxidative utilization, and multi-level impairment.

#### 3.4.1. Impaired Convective Oxygen Delivery

Reduced cardiac output was identified as the primary upstream constraint in CHF, congenital heart disease, and pulmonary arterial hypertension. In CHF, prolonged phase I duration and slowed phase II time constants of pulmonary VO_2_ kinetics reflect impaired bulk oxygen delivery, with patients exhibiting greater reliance on fractional oxygen extraction at submaximal work rates due to reduced blood flow capacity [68]. A single NIRS-based investigation in this review reported that hyperoxia increased peak VO_2_ in CHF patients, suggesting that convective and diffusive oxygen delivery, rather than mitochondrial capacity, was the proximal limit to performance in that cohort [68]. However, the broader CHF literature documents substantial skeletal muscle mitochondrial dysfunction, including reduced citrate synthase and electron transport chain enzyme activities, lower mitochondrial volume density, and impaired respiratory function [89,90,91], such that the relative contributions of delivery and mitochondrial impairment likely vary with disease severity, ambulatory status, and deconditioning and should not be inferred from a single NIRS-derived observation. In CHF patients with inspiratory muscle weakness, respiratory muscle fatigue triggers a sympathetically mediated metaboreflex that further redistributes blood flow away from locomotor muscles, compounding the delivery deficit [70].

A similar competition for cardiac output between respiratory and locomotor muscles has been documented in COPD, where the respiratory muscles may consume up to 50% of total oxygen uptake during exercise, and unloading the respiratory muscles with proportional assist ventilation improves leg muscle oxygenation without changing systemic oxygen delivery [32]. In adults with complex congenital heart disease, reduced cardiac output capacity, particularly in Fontan physiology where the absence of a subpulmonary ventricle limits exercise cardiac output augmentation, results in lower resting muscle oxygen saturation, slower desaturation at exercise onset, and slower post-exercise resaturation compared to controls [14].

Notably, fractional oxygen extraction did not differ between CHD patients and controls, indicating the impairment is primarily delivery driven [14]. In children with CHD, similar patterns of impaired respiratory and peripheral muscle oxygenation were observed, with training-induced improvements in respiratory muscle oxygenation correlating strongly with improvements in VO_2_ [75]. In pulmonary arterial hypertension, NIRS-derived tissue oxygenation index correlated significantly with invasive mixed venous oxygen saturation both at rest (r = 0.701) and during exercise (r = 0.863), and decreased significantly during exercise, reflecting progressive oxygen delivery–utilization mismatch as cardiac output fails to meet peripheral metabolic demand [71].

#### 3.4.2. Impaired Microvascular Distribution and Diffusive Transport

Peripheral artery disease represents the prototypical condition of macrovascular and microvascular oxygen delivery impairment. The functional limitation in PAD cannot be explained solely by arterial stenosis, as only moderate associations exist between the ankle–brachial index and maximal walking capacity [38]. Beyond macrovascular obstruction, endothelial dysfunction, impaired collateral flow, capillary rarefaction, and reduced microvascular reactivity all contribute to the oxygen supply–demand mismatch during exercise [23,49,76]. NIRS studies consistently demonstrate that PAD patients reach lower tissue oxygen saturation values during exercise, exhibit more abrupt desaturation at exercise onset, and have prolonged recovery times compared to healthy controls [49,58,61]. Revascularization primarily improves oxygen supply (oxygenated hemoglobin), whereas exercise training produces relatively greater improvements in oxygen utilization efficiency (deoxygenated hemoglobin), illustrating the dual supply-side and demand-side nature of the impairment [48].

In type 2 diabetes, NIRS revealed a transient overshoot of deoxygenated hemoglobin above steady-state levels in the first 90–100 s of exercise, indicating that microvascular blood flow increases too slowly relative to metabolic demand [52]. The estimated microvascular blood flow mean response time was significantly prolonged (47.7 ± 14.3 s vs. 35.8 ± 10.7 s in controls), attributed to impaired nitric oxide-dependent endothelial function, reduced capillary density, and altered basement membrane structures, while cardiac output during submaximal exercise appeared normal [52]. In type 1 diabetes, vascular dysfunction manifested as endothelial dysfunction, reduced arterial compliance, and decreased capillary-to-fiber ratio contributed to impaired muscle oxygen delivery, with a single small study (*n* = 16) reporting that one year of individualized training did not produce a measurable improvement in local muscle oxygen extraction despite equivalent improvements in VO_2_peak [57]. The modest sample size of this study limits statistical power to detect small to moderate training effects on NIRS-derived extraction, and this finding should be interpreted as a non-detection rather than as evidence of true absence of effect; replication in larger T1D cohorts is required to determine whether muscle oxygen extraction is genuinely resistant to training in this population.

In chronic kidney disease, microvascular hyperemic responses were progressively blunted with advancing CKD stages following a significant linear trend, attributed to endothelial dysfunction occurring early in CKD and deteriorating with disease progression [79]. Wavelet analysis of resting NIRS signals further revealed impaired neurogenic and endothelial regulation of microvascular blood flow in CKD, detectable even without exercise or occlusion provocations [82].

#### 3.4.3. Impaired Oxidative Utilization

In COPD, despite central delivery constraints, intrinsic skeletal muscle dysfunction plays a substantial and independent role. Using neuromuscular electrical stimulation to bypass central cardiorespiratory limitations, de Paiva Azevedo et al. demonstrated markedly slower HHb recovery time constants in COPD patients (23.0 ± 7.5 s vs. 11.0 ± 6.8 s at 40 mA), with elevated microvascular oxygen extraction fraction even when corrected for muscle mass and work output, while total hemoglobin changes remained similar between groups [60]. These findings implicate reduced mitochondrial electron transport chain components, impaired biogenesis, a shift from oxidative type I to glycolytic type II fibers, decreased oxidative enzyme activity, and reduced capillary density as contributing peripheral mechanisms [60,77]. Notably, when arterial oxygen availability was preserved through supplemental oxygen, the rate of muscle desaturation per unit work was nearly identical between COPD patients and controls, suggesting that skeletal muscles retain their functional oxygen utilization capacity when freed from central constraints [81]. The spatial heterogeneity of muscle blood flow and metabolism within the vastus lateralis increased with exercise intensity in COPD patients, reflecting disturbed capillary-to-fiber interface and fiber type shifts, yet the VO_2_/Q ratio remained tightly matched across regions, indicating a preserved autoregulatory compensatory mechanism [74]. In metabolic myopathies, genetic mutations directly impair mitochondrial respiratory chain function, producing markedly lower peak fractional oxygen extraction, transient deoxygenation overshoots, a 30–40% higher oxygen cost of exercise, and slower VO_2_ kinetics [86]. In a single small study of women with breast cancer treated with adjuvant chemotherapy (*n* = 32), muscle microvascular toxicity, cachexia-induced wasting, and treatment-related mitochondrial myopathy were proposed to collectively impair oxygen utilization [43]. This single-study evidence cannot support generalization to the broader cancer survivor population, which encompasses heterogeneous tumor types, chemotherapy regimens, and post-treatment intervals; NIRS-based characterization of muscle oxygenation in cancer survivors is an emerging area requiring further research across cancer types and treatment regimens before population-level conclusions can be drawn.

#### 3.4.4. Multi-Level Impairment

Several populations exhibit impairment at multiple levels of the oxygen cascade simultaneously. In COPD, both slowed cardiac output kinetics and accelerated muscle deoxygenation kinetics at exercise onset indicate concurrent central delivery and peripheral utilization deficits, with the ratio of VO_2_ kinetics to HHb kinetics (τVO_2_p/MRT-[HHb]) nearly double in COPD patients compared to controls (4.9 ± 1.2 vs. 2.5 ± 0.6) [37]. In PAD, both supply-side deficits (arterial stenosis, impaired collateral flow) and demand-side inefficiency (mitochondrial dysfunction, reduced oxidative enzyme activity) coexist, with each component responding differently to revascularization versus exercise training [48,76]. In CKD, the progressive deterioration of microvascular reactivity is compounded by myosteatosis and myofibrosis, with intramuscular fat infiltration and fibrotic tissue accumulation further impairing capillary-to-fiber oxygen diffusion and reducing contractile tissue proportion [87]. The entropy analysis of NIRS signals in type 2 diabetes revealed a distinctly different muscular metabolic pattern compared to healthy controls, with lower signal complexity indicating impaired neuromuscular recruitment and peripheral vascular insufficiency, a pattern that normalized after one year of exercise [69].

### 3.5. Diagnostic Assessment of Muscle Oxygenation

Across the included studies, 12 non-intervention investigations characterized population-specific NIRS signatures of muscle oxygenation impairment, contributing the core observational basis for the diagnostic synthesis presented in this section. In peripheral artery disease, the dominant signature was prolonged reoxygenation, with 50% StO_2_ recovery time (T50) extending well beyond healthy reference values and discriminating affected from unaffected limbs [58,61,76]. In chronic heart failure, the characteristic signature was slowed phase II VO_2_ kinetics coupled with reduced peak deoxygenation amplitude, consistent with impaired oxygen delivery during the transient [68]. In type 2 diabetes, a transient deoxygenation overshoot at exercise onset was observed across multiple cohorts, indicating delivery–utilization mismatch [52,69]. In chronic kidney disease, blunted post-occlusion hyperemic responses progressed with advancing disease stage and were accompanied by impaired neurogenic and endothelial microvascular regulation on resting frequency-domain analysis [79,82,87]. In chronic obstructive pulmonary disease, resting tissue hypoxia and impaired Δ[HHb]/ΔVO_2_ matching during incremental exercise distinguished affected patients from controls [74,81]. In congenital and pediatric heart disease, reduced peak deoxygenation amplitude during incremental exercise paralleled reductions in peak VO_2_ [14,75]. Each of these signatures is integrated below into the broader diagnostic synthesis organized by testing protocol; population-specific reference findings and supporting effect sizes from individual studies are detailed in Table 1.

#### 3.5.1. Exercise-Based Protocols

Incremental or ramp protocols (n = 21) characterized the progressive relationship between oxygen demand and supply, while constant-load protocols (n = 12) evaluated oxygen delivery–utilization kinetics during exercise transitions and recovery. Key diagnostic variables included peak deoxygenation amplitude, desaturation rate, recovery time constants (τHbO_2_), reoxygenation half-time (T50), and the deoxygenation-to-VO_2_ ratio (Δ[HHb]/ΔVO_2_) as an index of microvascular delivery–utilization matching [37,50,56], with a transient deoxygenation overshoot indicating delivery–utilization mismatch [52,86]. The NIRS-derived reoxygenation time constant correlated significantly with citrate synthase activity from muscle biopsies at the same site, directly validating it as a non-invasive proxy for muscle oxidative enzyme capacity [77]. In PAD, the foundational feasibility study by Comerota and colleagues reported that 50% calf StO_2_ recovery times exceeding 70 s discriminated affected from unaffected subjects with 89% sensitivity and 85% specificity in a single-center cross-sectional cohort of 49 participants using the InSpectra tissue spectrometer [92]. Subsequent PAD studies have confirmed prolonged StO_2_ recovery times in affected versus unaffected limbs across independent cohorts [38,58,61], but prospective external validation of the T50 > 70 s threshold across independent populations, harmonized exercise protocols, and the full range of contemporary NIRS devices has not yet been undertaken. In pulmonary arterial hypertension, NIRS-derived StO_2_ correlated strongly with invasive mixed venous oxygen saturation at rest (r = 0.701) and during exercise (r = 0.863), supporting its potential as a non-invasive surrogate for central hemodynamic monitoring [71].

Despite the diagnostic variables enumerated above, no exercise-NIRS test has yet been formally validated against a clinical reference standard or assessed by ROC analysis with population-specific threshold derivation, except in PAD. This represents the principal diagnostic translation gap identified by this review and is revisited in Section 4.3.

#### 3.5.2. Vascular Occlusion Protocols

Eleven studies incorporated arterial or venous occlusion maneuvers for physiological calibration and direct assessment of microvascular reactivity [34,45,46,49,58,63,68,76,79,86]. In chronic kidney disease, occlusion–reperfusion testing revealed progressively blunted hyperemic responses across advancing stages [79]. Combining NIRS with diffuse optical spectroscopy during occlusion provided a model-based estimate of resting muscle oxygen consumption in PAD [46], while broadband NIRS during occlusion provided a direct non-invasive marker of mitochondrial function via the cytochrome c oxidase oxidation state [45]. The total labile signal calibration technique was applied in pediatric congenital heart disease to standardize cross-individual comparisons [65]. Across these protocols, calibration approaches varied substantially, and standardized occlusion parameters (cuff pressure, occlusion duration, time-to-peak window) have not been established for cross-study comparison.

#### 3.5.3. Emerging Approaches

Beyond exercise and occlusion protocols, several studies explored novel diagnostic paradigms. Resting NIRS measurements detected oxygenation abnormalities without exercise provocation, expanding diagnostic utility to populations for whom exercise is impractical; in COPD, resting SmO_2_ identified patients with tissue-level hypoxia (40–57%), with rehabilitation shifting 16 of 40 patients to normoxic levels [78], while wavelet time–frequency analysis of resting NIRS recordings in CKD revealed impaired neurogenic and endothelial microvascular regulation [82]. Spatial heterogeneity of deoxygenation across the vastus lateralis was mapped using multi-channel systems, revealing non-uniform oxygen supply–demand matching not captured by single-site measurements [64,73,74]. Entropy-based analysis distinguished diabetic from healthy metabolic patterns [69], and frequency-domain NIRS combined with electrical stimulation determined voluntary versus electrically evoked oxygen demand to reveal age-related differences in muscle composition [47]. These emerging approaches collectively demonstrate the analytical depth attainable from NIRS signals beyond simple amplitude metrics, but none have yet been validated as a standardized clinical test, and the absence of published normative reference ranges constrains their immediate diagnostic application.

### 3.6. Exercise-Based Interventions Targeting Muscle Oxygenation

**Figure 5:**
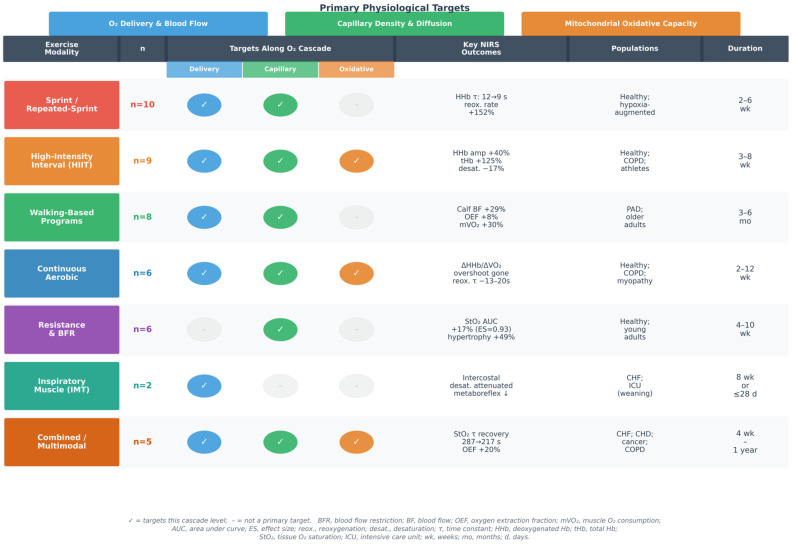
Exercise-based interventions targeting muscle oxygenation. Rows list exercise modalities; the three target columns indicate which step of the oxygen cascade each modality primarily addresses. A check mark (✓) indicates the modality targets that cascade level; an en dash (–) indicates it is not a primary target. The colored band groups the cascade targets into oxygen delivery and blood flow, capillary density and diffusion, and mitochondrial oxidative capacity. BFR, blood flow restriction; BF, blood flow; OEF, oxygen extraction fraction; mVO_2_, muscle O_2_ consumption; AUC, area under curve; ES, effect size; reox., reoxygenation; desat., desaturation; τ, time constant; HHb, deoxygenated Hb; tHb, total Hb; StO_2_, tissue O_2_ saturation; ICU, intensive care unit; wk, weeks; mo, months; d, days.

The exercise modalities identified across 49 intervention studies, their primary physiological targets along the oxygen transport cascade, key NIRS-derived outcomes, and training parameters are summarized in Figure 5, including sprint and repeated-sprint training, high-intensity interval training, walking-based programs, continuous aerobic and endurance training, resistance training and blood flow restriction, inspiratory muscle training, and combined and multimodal programs.

#### 3.6.1. Sprint and Repeated-Sprint Training

Sprint-based protocols consistently enhanced muscle oxygen extraction and reoxygenation kinetics. Two weeks of repeated Wingate sprint training accelerated HHb kinetics during both moderate and severe exercise (τ reduced from 12 to 9 s), increased deoxygenation amplitude, and improved exercise tolerance by 53%, with changes in VO_2_ kinetics strongly correlated with HHb adaptations (r = 0.71–0.81) [31]. Endurance training incorporating interval sprints improved post-sprint reoxygenation rate by 152%, correlating with improved repeated-sprint performance [35]. When sprint training was performed under hypoxic conditions, augmented muscle perfusion and deoxygenation adaptations were observed compared to normoxic training. In cross-country skiers, repeated-sprint training in hypoxia increased total hemoglobin oscillations approximately threefold and deoxyhemoglobin amplitude by 225%, compared to twofold and a 27% decrease in normoxia [40]. Similar hypoxia-augmented deoxygenation and reoxygenation responses were reported in team-sport athletes [41,72], with improvements linked to elevated HIF-1α and VEGF suggesting angiogenic mechanisms [72]. Voluntary hypoventilation during repeated sprints produced lower maximal deoxygenation and enhanced reoxygenation between sprints, accompanied by improved potassium handling and fatigue resistance [66].

#### 3.6.2. High-Intensity Interval Training

HIIT improved both muscle oxygenation responses and aerobic capacity across populations. Six weeks of aerobic interval training in healthy subjects reduced the deoxygenation slope by 16.6% while increasing HHb amplitude by 40.4% and total hemoglobin by 125.3%, with changes significantly correlated with VO_2_peak improvement [36]. In COPD, 8 weeks of supervised interval cycling amplified exercise-induced StO_2_ desaturation and post-exercise rebound in both patients and healthy controls, consistent with enhanced muscle oxygen transfer and utilization [24]. Unilateral knee-extension HIIT in hypoxia increased muscle blood volume oscillations during sprints by 80%, though performance gains were similar between hypoxic and normoxic conditions [39]. Adding post-exercise blood flow restriction (BFR) to sprint interval training increased peak fractional oxygen extraction (from 57.3% to 62.0%) and decreased TOI nadir at exhaustion, effects not seen with SIT alone [34]. Similarly, HIIT with BFR in endurance athletes attenuated maximal deoxygenation during Wingate testing while improving power output, despite 31% lower training loads [33]. However, six weeks of HIIT did not reduce the spatial heterogeneity of muscle deoxygenation in healthy untrained men, suggesting that intramuscular delivery–utilization matching may require longer or different training stimuli [73].

#### 3.6.3. Walking-Based Programs

In PAD, walking-based training consistently improved functional capacity through peripheral adaptations rather than increased arterial inflow. Supervised treadmill training increased maximal calf muscle blood flow by 29% and oxygen extraction fraction by 8%, without changes in ankle–brachial index or resting perfusion [23]. Three months of combined supervised and home-based walking increased exercise tolerance by 56% with greater calf deoxygenation during longer exercise, interpreted as enhanced capillarization and arteriovenous extraction [61]. Diffuse optical spectroscopy revealed that training increased resting muscle metabolic rate of oxygen by 30%, driven predominantly by enhanced oxygen extraction rather than blood flow [46]. A hybrid walking program in intermittent claudication produced slower deoxygenation during exercise and faster reoxygenation during recovery [38].

Both conventional and weighted walking improved StO_2_ recovery and walking economy, with conventional training showing greater deoxygenation rate improvement [49]. Revascularization primarily improved oxygen supply (oxygenated hemoglobin), while exercise training produced proportionally greater improvements in oxygen utilization [48]. NIRS-guided exercise training, using a 15% tissue desaturation threshold to modulate intensity, achieved comparable improvements to traditional pain-guided training [76]. Walking training at low intensity (~40% VO_2_max) in older women improved the microvascular oxygen delivery–utilization ratio (Δ[HHb]/ΔVO_2_) from 1.32 to 1.07 without changes in VO_2_max [54].

#### 3.6.4. Continuous Aerobic and Endurance Training

Endurance cycling training improved oxygen delivery–utilization matching across age groups. In both young and older men, 12 weeks of cycling at 70% VO_2_peak attenuated the transient overshoot in the Δ[HHb]/ΔVO_2_ ratio, with changes highly correlated with faster VO_2_ kinetics (r = 0.93–0.98), though the overshoot persisted in older men [50]. A single small short-term training study in young healthy men (*n* = 12) reported that one to two training sessions were sufficient to reduce the delivery–utilization mismatch, with adaptations persisting through 14 days of detraining [56]; given the modest sample size, this rapid-onset finding should be regarded as preliminary pending replication in larger cohorts and in clinical populations. Six weeks of endurance cycling enhanced mean muscle deoxygenation at peak exercise without altering spatial heterogeneity across the vastus lateralis [64]. Short-term HIIT and continuous training produced equivalent acceleration of VO_2_ kinetics (~40% after 8 sessions) with unchanged deoxygenation kinetics, suggesting concurrent microvascular perfusion adaptations that maintained delivery–utilization matching [67]. In COPD, 6 weeks of high-intensity cycling reduced the reoxygenation time constant by 13–20 s across exercise intensities, with improvements correlated with citrate synthase activity from muscle biopsies at the same site [77]. In metabolic myopathies, 12 weeks of moderate-intensity cycling increased peak fractional oxygen extraction by 50–100%, virtually eliminated the transient deoxygenation overshoot, and reduced the oxygen cost of exercise [86].

#### 3.6.5. Resistance Training and Blood Flow Restriction

Velocity-based squat training at lower fatigue levels (20% velocity loss) improved tissue oxygenation during exercise and recovery, while higher-fatigue training (40% velocity loss) did not [59]. Partial range of motion resistance exercise sustained greater intramuscular hypoxia than full range, correlating with superior hypertrophic responses (48.7% vs. 28.2% CSA increase) [42].

Blood flow restriction was investigated across heterogeneous protocols and populations. In healthy young men, low-load resistance training with BFR enhanced microvascular function (17% increase in StO_2_ AUC during reactive hyperemia vs. 5% with heavy loads) [63] but impaired endurance adaptations when applied to failure [44]; heavy-load resistance with BFR produced individualized hypertrophy patterns without consistent advantage over heavy load alone [88]. As an add-on, BFR augmented peak fractional oxygen extraction when paired with sprint interval training [34], and attenuated maximal deoxygenation despite 31% lower training loads when paired with HIIT [33]. In heart failure with reduced ejection fraction, a single non-randomized trial of heavy-load resistance with BFR reported improvements in muscle oxygenation alongside functional gains [25]. Given the small number of studies and the heterogeneity of populations and protocols, the present synthesis does not support generalization about BFR training as a single intervention category; population- and protocol-specific evidence is needed.

#### 3.6.6. Inspiratory Muscle Training

Inspiratory muscle training was investigated in three distinct clinical contexts, each requiring separate interpretation. In ambulatory CHF outpatients with inspiratory muscle weakness, 8 weeks of home-based IMT at 30% of maximal inspiratory pressure attenuated oxygen desaturation in both intercostal and forearm muscles during respiratory fatigue, providing mechanistic evidence for attenuation of the inspiratory muscle metaboreflex [70]. In a separate population of mechanically ventilated ICU patients with weaning difficulties, daily high-intensity IMT over up to 28 days improved scalene and sternocleidomastoid oxygenation parameters compared to sham training, including enhanced oxygen delivery, utilization, and blood volume [80]; the very different clinical context (acute critical illness with mechanical ventilation versus stable outpatient CHF) precludes pooling of these results into a single inference about IMT efficacy. In healthy adults, respiratory muscle training (both voluntary isocapnic hyperpnea and inspiratory threshold loading) reduced intercostal deoxygenation during exercise but did not alter vastus lateralis oxygenation, indicating localized rather than systemic effects [53].

#### 3.6.7. Combined and Multimodal Programs

In heart failure, 3 months of interval rehabilitation reduced the StO_2_ recovery time constant from 287 to 217 s [85], while 12 weeks of combined endurance and resistance training decreased peak exercise hemoglobin concentrations and sympathetic nerve activity alongside improved blood flow and VO_2_peak [25]. Light-to-moderate-intensity training in CHF increased peak fractional oxygen extraction by 20% and reduced VO_2_ kinetics mean response time by 19%, with simultaneous increases in circulating endothelial progenitor cells [68].

In children with CHD, 12 weeks of home-based interval cycling improved respiratory muscle oxygenation from 60% VO_2_max onward, with changes strongly correlated with VO_2_ improvements (r = 0.90) [75], and peripheral muscle oxygenation recovery was accelerated alongside improved endurance (r = 0.90) [55]. Home-based resistance training in pediatric CHD produced medium-effect reductions in TOI at peak exercise (d = 0.67), suggesting enhanced oxygen extraction [65]. In a single trial of multimodal training during chemotherapy in women with breast cancer (*n* = 32), muscle deoxygenation increased by 51.7% (ES = 0.69), accompanied by improved strength and endurance [43].

Four weeks of inpatient pulmonary rehabilitation in COPD shifted 16 of 40 patients from hypoxic to normoxic resting SmO_2_ levels, with significant improvements in functional outcomes [78]. A one-year exercise program normalized NIRS entropy patterns in type 2 diabetes, making previously distinguishable metabolic signatures indistinguishable from healthy controls [69]. One-leg cycling during ACL rehabilitation maintained VO_2_peak and improved vastus lateralis oxygenation and blood volume at all submaximal intensities, preventing the deconditioning observed in controls [51]. Interval training during limb unloading preserved deoxygenation kinetics (τ unchanged), while controls showed significant deterioration [62]. In adults with type 1 diabetes (*n* = 12), one year of individualized training improved VO_2_peak equivalently to matched controls, but peak muscle deoxygenation increased only in controls [57]. A plausible but unproven mechanism for this apparent non-response involves chronic hyperglycemia-induced glycation of hemoglobin, which alters the oxygen–hemoglobin dissociation curve and may compromise tissue oxygen unloading at the microvascular step; this mechanism was not directly demonstrated by the cited study and is presented as a hypothesis for future testing rather than as an established finding.

### 3.7. Certainty of Evidence Across Exercise Modalities

**Figure 6:**
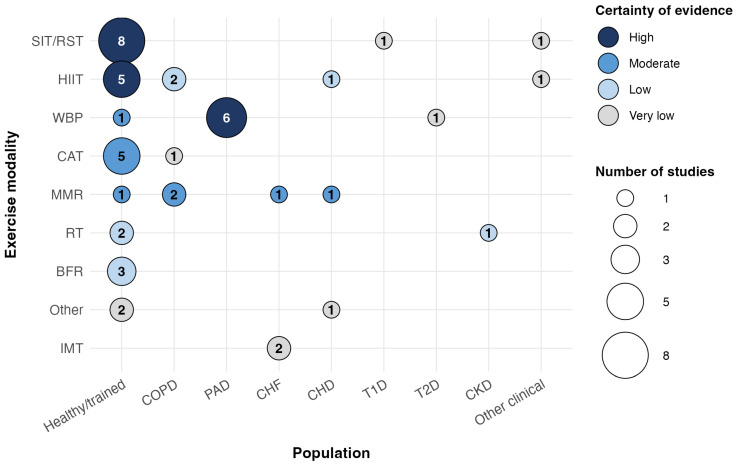
Population-stratified evidence map of exercise modalities targeting muscle oxygenation.

The evidence base across the seven modalities described in Section 3.6 was uneven in both study number and effect consistency (Table 3, Figure 6). Certainty ratings in this review were derived from four pragmatic criteria: number of contributing studies, consistency of effect direction, study quality (Section 3.2), and mechanistic plausibility based on reported physiological outcomes. The formal GRADE framework was not applied because (i) GRADE is designed for outcome-specific certainty rating in the context of meta-analysis, which was not performed here; and (ii) the heterogeneity of NIRS outcomes across studies precluded a single common outcome metric on which to apply GRADE. The four criteria broadly correspond to GRADE domains: number of studies and consistency map to imprecision and inconsistency; study quality maps to risk of bias; and mechanistic plausibility maps to indirectness (in reverse) as well as to biological gradient as an upgrading factor. Publication bias was not formally assessed and is addressed as a limitation in Section 4.5. Certainty was rated separately for healthy/trained and clinical populations because the evidence distribution differed substantially between subgroups (Table 3).

In healthy and trained populations, high certainty was observed for sprint/repeated-sprint training (*n* = 10) [31,33,35,39,40,41,42,43,57,83] and HIIT (*n* = 9) [24,34,36,38,49,51,53,63,67], each supported by multiple independent studies with consistent effect direction; moderate certainty was observed for continuous aerobic training (*n* = 5) [50,56,64,67]; and low certainty was observed for resistance training (*n* = 2) [44,45] and blood flow restriction (*n* = 5) [33,34,44,63,88], where evidence was limited or protocols were heterogeneous. In clinical populations, high certainty was observed only for walking-based programs in PAD (*n* = 8) [23,46,47,48,52,58,61,86]; moderate certainty for combined multimodal rehabilitation in mixed populations (*n* = 5) [25,55,60,71,72]; and low-to-very-low certainty for all other modalities, including HIIT (*n* = 3; PAD, COPD) [38,53,66], continuous aerobic training (*n* = 1) [85], resistance training (*n* = 1; CKD) [87], inspiratory muscle training (*n* = 3; CHF outpatient, ICU, healthy adults) [53,70,80], and a single null sprint training study in type 1 diabetes [57]. No modality reached high certainty in populations with predominantly reduced convective oxygen delivery (CHF, CHD, pulmonary arterial hypertension), and high-certainty modality–population pairings were limited to sprint and HIIT for peripheral oxidative adaptation in healthy and trained subjects, and walking-based programs in PAD. The substantial asymmetry between healthy/trained and clinical certainty levels should inform clinical recommendations, as the magnitude and mechanism of training-induced muscle oxygenation adaptations in clinical populations cannot be inferred from evidence developed in healthy/trained cohorts, and population-specific trials remain a research priority.

## 4. Discussion

### 4.1. Summary of Principal Findings

This systematic review synthesized 61 studies published between 2003 and 2025 that used NIRS to characterize muscle oxygenation in healthy and pathophysiological conditions, encompassing 27 RCTs, six NRCTs, 17 pre–post intervention studies, and 10 cross-sectional or observational investigations. Common pathophysiological processes affecting muscle oxygenation regulations include COPD, PAD, CHF, CHD, and diabetes. Three principal findings emerge from the synthesis of the findings of the included studies.

First, muscle oxygenation impairment is best conceptualized not as a single pathology but as a cascade-level problem, with mechanistically distinct phenotypes corresponding to the dominant site of impairment along the oxygen transport pathway—convective delivery-dominant (CHF, CHD, pulmonary arterial hypertension), microvascular and diffusive-dominant (PAD, type 1 and type 2 diabetes, chronic kidney disease), oxidative utilization-dominant (COPD peripheral myopathy, metabolic myopathies, chemotherapy-related myopathy), and multi-level impairment phenotypes—each with characteristic NIRS signatures.

Second, although NIRS has matured into the most widely used non-invasive tool for assessing muscle oxygenation and demonstrated diagnostic value in specific contexts, including 50% StO_2_ recovery times exceeding 70 s for PAD diagnosis (89% sensitivity, 85% specificity, derived in a single-center feasibility cohort and awaiting external validation [92]) and strong correlations between NIRS-derived StO_2_ and invasive mixed venous oxygen saturation in pulmonary arterial hypertension, its clinical translation remains constrained by substantial methodological heterogeneity across devices, source–detector configurations, calibration procedures, adipose tissue correction, and outcome nomenclature.

Third, exercise-based interventions produce mechanistically distinct adaptations that align with the underlying cascade lesion, such that sprint and high-intensity interval training predominantly enhance peripheral oxidative capacity and reoxygenation kinetics; walking-based programs in PAD improve oxygen extraction and capillarization rather than arterial inflow; continuous aerobic training restores delivery–utilization matching; inspiratory muscle training attenuates the respiratory metaboreflex in CHF and COPD; and combined multimodal programs are most effective in populations with multi-level impairment.

Taken together, these findings support a unifying framework in which the site of cascade impairment determines both the appropriate diagnostic protocol and the optimal exercise prescription, an integrative perspective that has not been systematically developed in prior reviews and that forms the basis for the subsequent discussion.

### 4.2. Pathophysiology: A Cascade-Level Framework for Muscle Oxygenation Impairment

A unifying observation across the 61 included studies is that muscle oxygenation impairment is not a single pathological entity but a cascade-level phenomenon, in which the dominant site of impairment along the oxygen transport pathway determines both the NIRS signature at the bedside and the therapeutic response to intervention. The oxygen transport system operates as an integrated series of convective and diffusive steps, each of which contributes to overall capacity while none function as a single limiting factor [93]. This integrative view, together with the heterogeneity of NIRS findings across populations, motivates a phenotypic classification, in which patients are grouped not solely by primary diagnosis but by the dominant cascade lesion that explains their muscle oxygenation deficit. In this framework, each population is assigned to its primary cascade level based on the dominant NIRS signature across the included studies; populations with documented impairment at more than one level appear at each relevant level (Figure 4), reflecting concurrent multi-level involvement rather than mutually exclusive categorization. The primary assignment indicates the strongest empirical evidence within this review rather than the sole site of impairment, and the framework is offered as hypothesis-generating rather than as a validated clinical decision tool.

#### 4.2.1. Convective Delivery-Dominant Phenotype

The first group is characterized by impairment upstream of the muscle, at the level of ventilation, pulmonary diffusion, or cardiac output, with peripheral oxygen extraction largely preserved. In CHF, slowed VO_2_ kinetics and increased peak VO_2_ during hyperoxia localize the limitation upstream of the mitochondrion [68], and respiratory muscle weakness further redistributes blood flow away from locomotor muscles through a sympathetically mediated metaboreflex [70]. Adults with Fontan physiology show lower resting StO_2_, slower desaturation, and slower resaturation with unchanged fractional extraction, consistent with a delivery-driven lesion [14], and pulmonary arterial hypertension follows the same pattern, with NIRS-derived StO_2_ tracking invasive mixed venous oxygen saturation and decreasing as cardiac output fails to meet demand [71]. A parallel approach using invasive CPET with personalized O_2_ pathway analysis has rank-ordered cascade defects in HFpEF, providing quantitative support for the delivery-dominant interpretation developed here [94]. The clinical implication is that therapies augmenting convective delivery, whether through respiratory muscle offloading, cardiac output reserve, or inspiratory muscle training, are mechanistically aligned with the underlying lesion.

#### 4.2.2. Microvascular and Diffusive-Dominant Phenotype

The second group is defined by impairment at the microvascular and diffusive steps, with macrovascular delivery and mitochondrial capacity comparatively less affected. PAD is the prototypical example, where ankle–brachial index correlates only moderately with walking capacity [38], and endothelial dysfunction, capillary rarefaction, and reduced microvascular reactivity each contribute to the supply–demand mismatch [23,49,76]. The differential response to revascularization versus exercise training further underscores the dual supply-side and diffusive nature of the lesion [48]. In type 2 diabetes, the transient HHb overshoot and prolonged microvascular mean response time localize the impairment to impaired endothelial function and reduced capillary density despite normal cardiac output [52]; type 1 diabetes shows blunted local oxygen extraction even after successful improvements in VO_2_peak [57]. In CKD, progressively blunted hyperemic responses and wavelet-derived evidence of impaired neurogenic and endothelial regulation confirm a predominantly microvascular lesion [79,82]. The shared clinical implication is that ankle–brachial index or resting perfusion underrepresents the functional deficit, and therapies targeting microvascular reactivity are more likely to succeed than those targeting bulk flow.

#### 4.2.3. Oxidative Utilization-Dominant Phenotype

The third group is distinguished by intrinsic skeletal muscle dysfunction, in which mitochondrial oxidative capacity constrains muscle VO_2_, even when delivery is adequate. In COPD, neuromuscular electrical stimulation bypassing central cardiorespiratory limits reveals markedly slower HHb recovery and elevated extraction fraction consistent with reduced electron transport chain capacity, type I-to-II fiber shift, and decreased oxidative enzyme activity [60,77]. Conversely, when arterial oxygen is preserved with supplemental oxygen, muscle desaturation per unit work is nearly identical to controls, demonstrating that the peripheral apparatus retains functional capacity when freed from central constraints [81]. These observations align with the ATS/ERS consensus that limb muscle dysfunction is a key systemic consequence of COPD independent of airway disease severity [95]. Metabolic myopathies produce analogous signatures, with markedly lower peak extraction, deoxygenation overshoots, and higher oxygen cost of exercise [86]; preliminary evidence from a single study in chemotherapy-treated breast cancer survivors suggests qualitatively similar findings [43], although the latter requires replication across cancer types and treatment regimens before classification within this phenotype is established. The clinical implication is that central interventions alone are insufficient, and peripheral stimuli capable of inducing mitochondrial remodeling are required.

#### 4.2.4. Multi-Level Impairment

The fourth group exhibits concurrent impairment at multiple cascade steps. COPD, despite its characterization above as oxidative utilization-dominant, also exhibits central delivery deficits, with τVO_2_p/MRT-[HHb] nearly double that of controls [37]; when ventilatory limits are minimized using small muscle mass exercise, both convective and diffusive muscle O_2_ transport are reduced by approximately 36% in COPD [96]. Advanced PAD combines supply-side deficits with demand-side inefficiency, each responding differently to revascularization versus training [48,76]. In CKD, microvascular deterioration is compounded by myosteatosis and myofibrosis that further impair capillary-to-fiber diffusion [87], and entropy analysis in type 2 diabetes reveals combined neuromuscular and vascular insufficiency that normalizes with one year of exercise [69]. Multi-level impairment is clinically important because single-target therapies are insufficient, and ratio-based and heterogeneity-based NIRS indices appear better suited to flagging these patients than any single kinetic parameter.

#### 4.2.5. Toward a Cascade Lesion Phenotyping Schema

**Figure 7:**
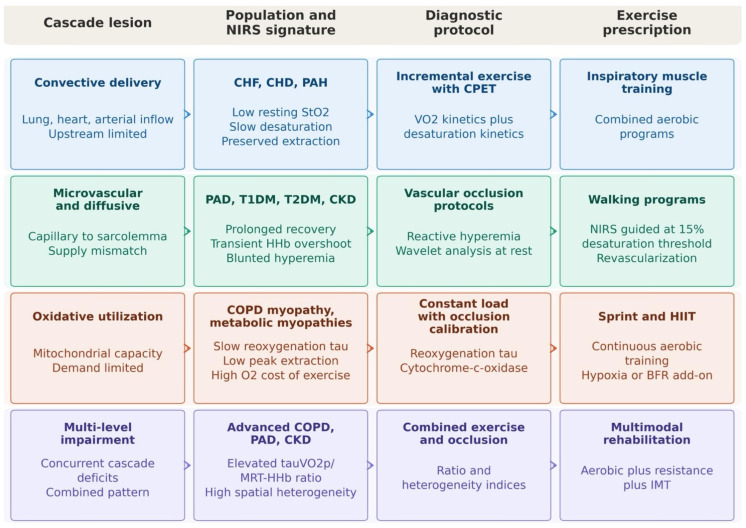
Integrated cascade lesion framework linking phenotype, diagnostic protocol, and exercise prescription. The framework is presented as a hypothesis-generating synthesis of the patterns observed across the included studies and is not a validated clinical decision tool. Operational application to individual patients requires prospective validation of population-specific NIRS thresholds, currently available only for selected conditions (notably PAD).

Taken together, the mechanistic diversity across the included studies supports a phenotyping approach, in which patients are classified by the dominant site of cascade impairment rather than by diagnosis alone. This proposal extends existing efforts to move beyond disease-label taxonomies. Personalized O_2_ pathway analysis in HFpEF has grouped patients by shared susceptibility to delivery-targeted versus extraction-targeted therapy [94], and phenotypic classification along a ventilatory-limited, cardiovascular-limited, and combined continuum has been proposed in COPD, acknowledging that a single diagnostic label can subsume mechanistically distinct exercise limitations [97]. The cascade lesion framework proposed here generalizes these approaches across populations, using the NIRS signature as the primary instrument for localizing the dominant lesion. Two features distinguish this schema from disease-by-disease taxonomy: a single diagnosis may encompass more than one phenotype, and a single phenotype may span diverse diagnoses. By making the cascade lesion rather than the diagnostic label the basis for classification, the schema directly informs the choice of diagnostic protocol (Section 4.3) and exercise prescription (Section 4.4) (Figure 7). Its clinical utility, however, depends on NIRS-derived signatures robust enough to discriminate between phenotypes in individual patients, a requirement that the methodological heterogeneity documented in Section 3.3 currently constrains. The framework is therefore offered as a hypothesis-generating construct rather than as a validated clinical decision tool, with prospective validation of population-specific NIRS thresholds (currently available only for selected conditions, notably PAD) representing a research priority before operational application to individual patients.

### 4.3. Diagnostic Assessment: Matching the Protocol to the Lesion

If the cascade lesion phenotyping schema proposed in Section 4.2 is to have clinical value, it must be paired with diagnostic protocols capable of localizing the dominant site of impairment in individual patients. The included studies employed three broad categories of NIRS-based assessment, each with distinct strengths and limitations that align differently with the four phenotypes described above. However, a critical gap between the diagnostic potential of NIRS and its clinical implementation persists, rooted not in the technology itself but in the methodological fragmentation documented in Section 3.3.

#### 4.3.1. Strengths of Current NIRS-Based Protocols

The three protocol categories identified across the included studies offer complementary windows into the oxygen transport cascade. Incremental and ramp protocols (*n* = 21) characterize the progressive relationship between oxygen demand and supply, revealing the global delivery–utilization matching profile and peak deoxygenation amplitude. Constant-load protocols (*n* = 12) resolve the temporal dynamics of oxygen delivery–utilization transitions, yielding kinetic parameters (τ, MRT, T50) and the transient deoxygenation overshoot that distinguishes microvascular-dominant and utilization-dominant phenotypes [37,50,52,56,86]. Vascular occlusion protocols (*n* = 11) provide direct assessment of microvascular reactivity and, when combined with advanced optical methods, enable quantification of resting muscle metabolic rate of oxygen [46] and mitochondrial function via cytochrome c oxidase oxidation state [45]. Emerging resting-state approaches, including wavelet time–frequency analysis [82] and entropy-based methods [69], extend diagnostic utility to patients for whom exercise provocation is impractical. Collectively, these protocols span the full range of cascade lesions, from upstream delivery deficits detectable by incremental exercise to intrinsic mitochondrial impairment assessable through occlusion-based techniques.

#### 4.3.2. Validated Diagnostic Thresholds: The Exception Rather than the Rule

Despite the breadth of available protocols, only a small number of studies have established quantitative diagnostic thresholds with reported sensitivity and specificity. In PAD, the foundational feasibility study by Comerota and colleagues reported 89% sensitivity and 85% specificity for a T50 > 70 s threshold in a single-center cross-sectional cohort using the InSpectra tissue spectrometer [92]; subsequent studies in independent PAD cohorts have confirmed prolonged StO_2_ recovery times in affected limbs [38,58,61], but prospective external validation of this specific threshold across independent populations and across the full range of NIRS devices has not yet been undertaken, and the result should be interpreted as a single-cohort derivation awaiting confirmation rather than as a generalized clinical diagnostic standard. In pulmonary arterial hypertension, NIRS-derived StO_2_ correlated strongly with invasive mixed venous oxygen saturation at rest (r = 0.701) and during exercise (r = 0.863), supporting its potential as a non-invasive hemodynamic surrogate [71]. In COPD, the NIRS-derived reoxygenation time constant correlated significantly with citrate synthase activity from muscle biopsies, directly validating it as a non-invasive proxy for muscle oxidative enzyme capacity [77]. These examples demonstrate that NIRS can achieve clinically meaningful diagnostic performance when paired with appropriate protocols and well-defined patient populations. However, they remain isolated benchmarks rather than components of a systematic diagnostic framework. The vast majority of included studies report correlations or group-level differences without deriving receiver operating characteristic curves, cutoff values, or prospective validation in independent cohorts. The absence of such thresholds for CHF, CHD, CKD, and diabetes represents a substantial gap that limits the clinical applicability of the cascade lesion phenotyping approach.

#### 4.3.3. Methodological Heterogeneity as the Field’s Central Bottleneck

The findings from Section 3.3 indicate that the barrier to clinical translation is not the absence of useful NIRS parameters but the lack of standardization in how those parameters are obtained and reported. Source–detector spacing ranged from 12.5 to 50 mm, sampling rates from 0.33 to 50 Hz, and adipose tissue thickness was measured in only 37% of studies, a notable gap given the known signal attenuation from subcutaneous fat. Calibration procedures varied from simple baseline referencing to physiological calibration using cuff occlusion, and tissue oxygen saturation was reported under at least five designations (StO_2_, TSI, TOI, SmO_2_, StiO_2_), reflecting device-specific nomenclature rather than distinct physiological constructs. This fragmentation has been recognized beyond the present review, as the Cores of Reproducibility in Physiology guidelines for NIRS highlighted the diversity of instrumentation, terminology, signal analysis, and lack of standardized protocols as key threats to the reproducibility of NIRS-based findings [8]; moreover, a recent updated systematic review of 191 muscle oximetry studies concluded that absolute values are generally not comparable between devices unless corrected by physiological calibration, and called for standardization using tissue-simulating phantoms and adherence to existing ISO guidelines [98,99]. These observations converge with our findings that cross-study comparisons are currently unreliable, and that diagnostic thresholds derived from one device-protocol combination may not transfer to another. Until minimum reporting standards are established, including mandatory disclosure of source–detector spacing, wavelength, sampling rate, calibration method, adipose tissue thickness, and outcome nomenclature, the translation of NIRS from a research tool to a standardized clinical test will remain stalled.

#### 4.3.4. Toward Matching the Diagnostic Protocol to the Suspected Cascade Lesion

Despite these limitations, the existing evidence supports a preliminary framework for selecting NIRS protocols based on the clinically suspected phenotype. When the primary suspicion is a convective delivery-dominant phenotype, as in CHF or CHD, incremental exercise with concurrent CPET [10] provides the most informative assessment, with NIRS-derived desaturation kinetics and VO_2_ kinetics jointly localizing the upstream limitation. When a microvascular-dominant phenotype is suspected, as in PAD, diabetes, or CKD, vascular occlusion protocols that quantify reactive hyperemia and recovery time constants offer the most direct window into the relevant lesion, supplemented by resting wavelet or entropy analyses where exercise is infeasible [69,79,82]. When an oxidative utilization-dominant phenotype is suspected, as in COPD peripheral myopathy or metabolic myopathies, constant-load protocols that resolve the transient deoxygenation overshoot and reoxygenation τ are best suited, ideally calibrated against physiological occlusion to enable cross-individual comparison [45,46,77]. For patients in whom multi-level impairment is likely, a combined approach incorporating both exercise-based and occlusion-based protocols may be necessary, with ratio-based indices (τVO_2_p/MRT-[HHb]) and spatial heterogeneity metrics providing the most discriminating information [37,64,74]. This matching framework is necessarily provisional, as it awaits prospective validation in studies that test whether protocol selection guided by the suspected cascade lesion improves diagnostic accuracy relative to a uniform one-size-fits-all approach. Nevertheless, it represents a logical extension of the phenotyping schema developed in Section 4.2 and provides a structured basis for future standardization efforts.

### 4.4. Exercise-Based Interventions: Matching the Prescription to the Phenotype

The cascade lesion phenotyping schema and diagnostic matching framework developed in Section 4.2 and Section 4.3 converge on a practical question: can exercise prescriptions be matched to the dominant cascade lesion to optimize therapeutic outcomes? The 49 intervention studies included in this review provide substantial, albeit not yet definitive, evidence that different exercise modalities produce mechanistically distinct adaptations along the oxygen transport cascade, and that these adaptations align with the phenotype they are best positioned to address.

#### 4.4.1. Modality–Mechanism Pairing Along the Oxygen Transport Cascade

Sprint and high-intensity interval training produced the most consistent peripheral oxidative adaptations, accelerating HHb kinetics, increasing deoxygenation amplitude, and improving reoxygenation rates, with changes strongly correlated with VO_2_ kinetics improvements [31,35,36]. These modalities are mechanistically aligned with the oxidative utilization-dominant phenotype, in which the primary deficit is mitochondrial capacity rather than upstream delivery. The addition of hypoxic conditions or post-exercise blood flow restriction further augmented peripheral deoxygenation and reoxygenation responses [33,34,40,41], suggesting that compounding the metabolic stimulus can amplify the adaptive signal at the muscle level. Walking-based programs in PAD improved functional capacity predominantly through enhanced oxygen extraction and capillarization rather than increased arterial inflow, with ankle–brachial index and resting perfusion unchanged despite substantial gains in exercise tolerance [23,61]. Revascularization, by contrast, primarily improved oxygenated hemoglobin [48]. This complementary rather than redundant relationship between exercise and revascularization is one of the clearest examples of how understanding the cascade lesion informs therapeutic strategy: the microvascular-dominant phenotype benefits from both supply-side restoration and demand-side training, each targeting a different step. Continuous aerobic training restored delivery–utilization matching as indexed by the Δ[HHb]/ΔVO_2_ ratio, with a small short-term study in healthy young men (*n* = 12) reporting rapid onset within one to two sessions and durability through 14 days of detraining [56]; as discussed in Section 3.6, this finding awaits replication in larger samples and in clinical populations, though the transient overshoot persisted in older men after 12 weeks [50], suggesting age-related limits to microvascular plasticity.

Inspiratory muscle training addressed a distinct mechanism, attenuating the respiratory metaboreflex that redistributes blood flow from locomotor to respiratory muscles in CHF [70] and improving respiratory muscle oxygenation in mechanically ventilated ICU patients [80]. This modality is specifically suited to the convective delivery-dominant phenotype in which respiratory muscle competition for cardiac output compounds the delivery deficit. Combined and multimodal programs were most effective in populations exhibiting multi-level impairment, with CHF patients showing simultaneous improvements in StO_2_ recovery, fractional extraction, and VO_2_ kinetics [25,68,85], as well as children with CHD showing correlated improvements in respiratory and peripheral muscle oxygenation [55,75].

#### 4.4.2. Dose–Response Considerations

**Figure 8:**
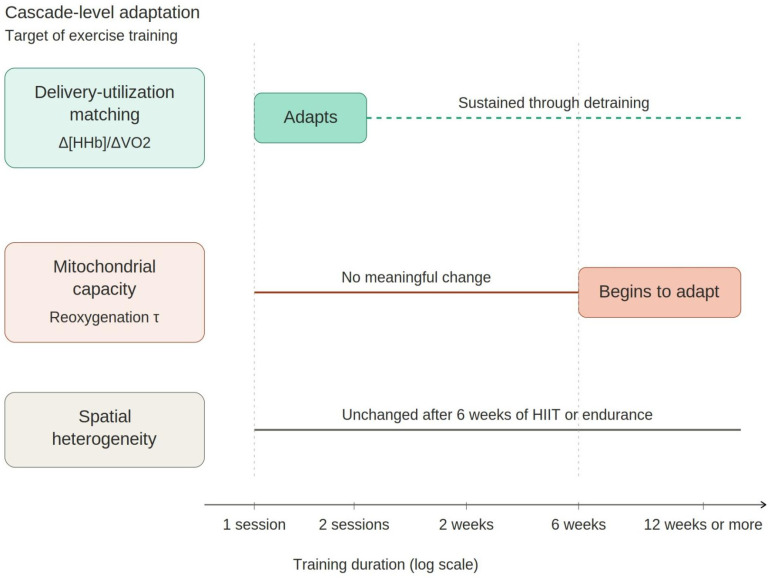
Differential time course of exercise-induced adaptations across the oxygen transport cascade.

Training durations across the included studies ranged from 2 weeks to over 1 year, with most programs lasting 4 to 12 weeks. The evidence suggests that different cascade targets require different temporal stimuli (Figure 8). Delivery–utilization matching, as reflected in the Δ[HHb]/ΔVO_2_ overshoot, has been reported to respond within one to two sessions in a small training study (*n* = 12) in healthy young men [56]; the rapid onset, if replicated, would be consistent with microvascular perfusion redistribution rather than structural remodeling. In contrast, mitochondrial adaptations, such as reoxygenation τ improvements, required 6 or more weeks and correlated with biopsy-confirmed increases in citrate synthase activity [77]. Spatial heterogeneity of deoxygenation, which may reflect capillary-level structural remodeling, did not change after 6 weeks of either HIIT or endurance training [64,73], suggesting that intramuscular delivery–utilization matching may require longer or qualitatively different stimuli. These observations imply that the expected time course of adaptation should be factored into both trial design and clinical expectations.

#### 4.4.3. NIRS as a Training Guidance Tool

Among the most clinically translatable findings was the use of NIRS-derived tissue desaturation thresholds to guide exercise intensity in PAD. Training guided by a 15% tissue desaturation threshold achieved comparable improvements to traditional pain-guided walking [76], offering an objective, reproducible alternative to a subjective symptom-based approach. This application exemplifies how NIRS can close the loop between diagnostic assessment and therapeutic prescription, where the same instrument that identifies the cascade lesion also monitors the adequacy of the training stimulus targeting that lesion. Although this approach has been tested only in PAD, its logic extends to any phenotype in which NIRS provides a real-time surrogate for the targeted adaptation.

#### 4.4.4. Non-Responders and Boundary Conditions

A single small study in adults with type 1 diabetes (*n* = 12) reported that 1 year of individualized training improved VO_2_peak equivalently to matched controls without a measurable increase in peak muscle deoxygenation [57]. This pattern, if replicated in larger cohorts, would illustrate a boundary condition in which a biochemical modifier (potentially the increased oxygen affinity of glycosylated hemoglobin) overrides the expected training response at the microvascular step; as discussed in Section 3.6, this mechanistic explanation is offered as a hypothesis for future testing and was not directly demonstrated by the included study. Similarly, high-fatigue resistance training (40% velocity loss) failed to improve tissue oxygenation despite comparable hypertrophic responses to lower-fatigue protocols [59], and low-load BFR impaired endurance adaptations relative to free-flow training, likely by limiting the aerobic metabolic stimulus necessary for oxidative remodeling [44,88]. These examples caution against assuming that greater training stress invariably produces greater oxygenation benefit and reinforce the principle that the prescription should be matched not only to the cascade lesion but also to the patient’s capacity to respond at that level.

### 4.5. Limitations of This Review

Several limitations should be acknowledged across the three domains addressed in this review.

Population representation was uneven, as COPD (*n* = 9) and PAD (*n* = 8) were well represented, but sarcopenia, frailty, aging-related deconditioning, and pediatric populations beyond congenital heart disease were underrepresented. Across the 61 included studies, 393 of 1403 participants with reported sex data (28.0%) were women, with substantial variation across populations (0% female in the single included type 1 diabetes study; 0–34% across PAD studies; 0–19% across COPD studies; 0–47% across CHF studies; 14–50% across CKD studies; 20.5% across healthy and trained cohorts; one breast cancer trial enrolled exclusively women). This systematic underrepresentation is methodologically consequential given documented sex differences in NIRS-derived microvascular responses, including slower desaturation and reperfusion rates, higher minimal tissue saturation, and lower hyperemic amplitude in women relative to men [100,101,102,103], as well as with sex differences in hemoglobin oxygen affinity and skeletal muscle capillary density. The clinical applicability of the cascade lesion phenotyping framework to women cannot be assumed without dedicated investigation, and deliberate recruitment of women across cascade phenotypes is identified as a research priority in Section 4.6. The cascade lesion phenotyping schema remains a hypothesis-generating framework rather than an operational clinical decision tool, pending prospective validation in individual patients.

Substantial heterogeneity in NIRS devices, source–detector configurations, calibration procedures, and outcome nomenclature precluded meta-analysis, and validated thresholds with reported sensitivity and specificity are currently available for only a small number of conditions, notably PAD. High non-reporting rates for technical parameters (36.1% for source–detector spacing; 32.8% for sampling rate; 62.3% for adipose tissue thickness) and the predominance of continuous-wave devices (88.5% of studies), which restricts absolute quantification of oxygen extraction fraction and muscle metabolic rate of oxygen to a minority of studies using frequency-domain or broadband systems, further constrain between-study comparability.

Training durations, modalities, and dose parameters varied considerably across the 50 intervention studies, precluding formal dose–response analysis, and phenotype-guided trials have not yet been conducted. Blinding is a particular limitation, as across the 27 RCTs, only 7.4% reported concealed allocation and 14.8% reported assessor blinding. Although double blinding is rarely feasible in exercise interventions, the near-universal absence of assessor blinding raises the possibility that NIRS-derived outcomes were interpreted with knowledge of group allocation. The directional consistency of effects across populations and modalities partially mitigates this concern but does not eliminate it.

The search included seven databases (Table S1); the gray literature, conference proceedings, dissertations, and non-English publications were not systematically searched. Formal assessments of publication bias (funnel plot, Egger’s test) were not performed because these methods are designed for quantitative meta-analysis on a common outcome metric and are not applicable to the narrative synthesis used here, where heterogeneity of NIRS outcomes precludes a single pooled estimate. Regarding the English-language restriction, records were not separately tagged by exclusion reason at the title and abstract stage, so a precise count of records excluded solely for language cannot be retrieved. Finally, the April 2026 search end date carries some risk of missing studies indexed in the database platforms after the search date.

### 4.6. Future Directions

The findings and limitations of this review suggest priority research directions in each of the three domains addressed here.

With regard to pathophysiology, the cascade lesion phenotyping schema proposed in Section 4.2 requires prospective validation as a classification tool in individual patients, with explicit testing of whether a cascade-based phenotype predicts clinical trajectory better than diagnostic label alone. Such validation, including derivation of population-specific NIRS thresholds for conditions beyond PAD, is a prerequisite before the framework summarized in Figure 7 can be applied operationally in clinical settings. Deliberate efforts to recruit women, older adults with multimorbidity, pediatric populations beyond congenital heart disease, and non-Western cohorts are needed to determine whether the phenotypes and their therapeutic implications generalize across demographics.

With regard to diagnostic assessment, the field most urgently requires consensus on minimum reporting standards for NIRS-based muscle oxygenation studies. A structured minimum reporting checklist organized by six domains (instrumentation, probe configuration, tissue corrections, calibration and analysis, outcome reporting, and test protocol) is proposed in Table 4, grounded in the Cores of Reproducibility in Physiology guidelines for NIRS [8] and the updated systematic review by Perrey et al. [98]. Adoption of this checklist would standardize disclosure of the parameters most consequential for cross-study comparison and would provide a concrete basis for editorial and reviewer assessment of NIRS-based manuscripts. Prospective studies are also needed to establish validated diagnostic thresholds with receiver operating characteristic analysis for each cascade lesion phenotype, extending beyond the current benchmarks available only for PAD. Emerging technologies, including wearable multi-channel NIRS, diffuse correlation spectroscopy, broadband cytochrome c oxidase measurement, and contrast-enhanced ultrasound [19,20,21], warrant head-to-head validation against established NIRS protocols to determine whether they offer incremental diagnostic value.

With regard to exercise-based intervention, phenotype-guided trials should test whether matching the exercise prescription to the dominant cascade lesion produces greater functional gains than standard care or a uniform one-size-fits-all program. Building on the 15% desaturation threshold concept demonstrated in PAD [76], NIRS-guided exercise prescription should be tested prospectively across additional populations to determine whether real-time biofeedback improves training outcomes relative to symptom-guided or heart rate-guided approaches.

## 5. Conclusions

This scoping review of 61 studies published between 2003 and 2025 synthesized the pathophysiology, diagnostic assessment, and exercise-based management of muscle oxygenation impairment across diverse clinical populations. Three conclusions emerge: First, muscle oxygenation impairment is a cascade-level phenomenon, in which the dominant site of impairment defines a mechanistically coherent phenotype that cuts across traditional diagnostic categories. Second, NIRS can localize the dominant cascade lesion through complementary exercise-based, occlusion-based, and resting-state protocols, but its translation into standardized clinical practice remains constrained by methodological heterogeneity and the scarcity of validated diagnostic thresholds beyond peripheral artery disease. In particular, the predominance of group-level statistical comparisons over patient-level ROC-based diagnostic threshold derivation is a critical gap that future studies should address. Third, exercise-based interventions produce mechanistically distinct adaptations that align with specific cascade lesions, supporting a phenotype-guided prescription.

Taken together, the site of cascade impairment links diagnosis to therapy and provides a foundation for individualized, mechanism-based rehabilitation. The proposed cascade lesion phenotyping schema is hypothesis-generating and requires prospective validation in individual patients. Future work should validate this schema, establish consensus on minimum reporting standards for NIRS-based research, derive diagnostic thresholds across cascade phenotypes, deliberately recruit underrepresented populations, and test whether phenotype-guided exercise prescription yields superior functional outcomes relative to uniform rehabilitation.

## Figures and Tables

**Table 1:** Characteristics of included studies.

Study	Design	Population (n, Sex, Age)	Health	Intervention	Duration/Freq	Comparator
Healthy/trained adults (n = 26)						
Bailey et al., 2009	RCT	RST: 5M/3F, 21 ± 4 y; ET: 5M/3F, 21 ± 4 y; CON: 5M/3F, 21 ± 4 y	Healthy	Wingate repeated-sprint trg (cyc.)	2 wk; 6 sess	Work-matched cont. cyc.; CON
Bergamasco et al., 2025	RCT	10M	Healthy	Heavy-load RT vs. heavy-load + BFR	10 wk; 2×/wk	HL-RT vs. HL-BFR
Bourgeois et al., 2025	RCT	18M, 24.9 ± 3.5 y	Healthy	Cycling HIIT + BFR vs. HIIT	3 wk; 3×/wk	HIIT without BFR
Breese et al., 2025	RCT	19M, 24 ± 5 y	Healthy	SIT vs. SIT + post-ex. BFR	4 wk; 2×/wk	SIT without BFR
Buchheit et al., 2011	Pre–post	18M, 34 ± 5 y	Healthy	Endurance trg prog.	8 wk; 2×/wk supervised + 1–2×/wk self	NA
Caen et al., 2019	Pre–post	11M, 21.8 ± 1.2 y	Healthy	Sup. interval cyc.	6 wk; 3×/wk	NA
Costilla et al., 2023	RCT	23M, 21.4 ± 2.4 y	Healthy	Vel.-based RT	6 wk; 2×/wk	Habitual activity
Espinosa-Ramírez et al., 2023	RCT	VIH: 6M/6F, 22 ± 1 y; ITL: 6M/6F, 21 ± 1 y	Healthy	IMT (IMT)	8 wk; VIH 3×/wk, ITL 5×/wk	VIH vs. ITL
Faiss et al., 2015	RCT	11M, 29.6 ± 8.8 y; 6F, 24.2 ± 5.0 y	Healthy	SkiErg sprint trg in hypoxia	2 wk; 3×/wk	HYP vs. NOR (RSH vs. RSN)
Furuichi et al., 2009	RCT	TR 6M, 21.0 ± 3.5 y; CON 5M, 22.4 ± 3.5 y	Healthy	Unilateral unloading + single-leg interval cyc.	20 d; every 2nd day	Unloading only (no interval trg)
Gatterer et al., 2018	RCT	TR: 6M, 21.0 ± 3.5 y; CON: 5M, 22.4 ± 3.5 y	Healthy	Cycling sprint trg in hypoxia	3 wk; 3×/wk	RSH vs. SIH
Goto et al., 2019	RCT	44M, 20–22 y	Healthy	RT: partial vs. full ROM	8 wk; 3×/wk	PRE vs. FRE
Hamasaki et al., 2018	Pre–post	20F, 68.5 ± 5.8 y	Healthy	Walking trg (WT)	12 wk; 3–4×/wk	NA
Horiuchi et al., 2023	RCT	24M, 22 ± 2 y	Healthy	RT: BFR vs. heavy-load	4 wk; 4 d/wk	HLRT (no BFR)
Ida et al., 2024	Crossover	Exp1: 8M, 26.9 ± 6.6 y; Exp2: 8M, 20.8 ± 3.1 y	Healthy	Low-load RT to failure + BFR	6 wk; 2×/wk	Free blood flow (FBF)
Jones et al., 2020	Pre–post	6M8F, 43.4 ± 6 y	Healthy	Marathon trg prog.	≥16 wk	NA
Kime et al., 2010	Pre–post	6M/3F, 27 ± 5 y	Healthy	Endurance cyc.	6 wk; 3 d/wk	NA
Lapointe et al., 2020	RCT	12M5F, 22.3 ± 1.2 y	Healthy	Repeated-sprint run. + VHL	4 wk; 2×/wk	VHL vs. CTL
Lin et al., 2023	Pre–post	Young: 7M/10F, 19.70 ± 1.59 y; Older: 7M/11F, 61.57 ± 5.02 y	Healthy	Sup. multi. ex.	12 wk; 2–3×/wk	NA
McKay et al., 2009	RCT	12M, 25 ± 4 y	Healthy	Cycling HIIT	19 d; 8 sess	HIT vs. END
Murias et al., 2010	Pre–post	Older: 8M, 68 ± 7 y; Young: 8M, 23 ± 5 y	Healthy	Endurance cyc.	12 wk; 3×/wk	NA
Murias et al., 2016	NRCT	12M, 24 ± 3 y	Healthy	Continuous cyc.	~2.5 wk; 8 sess	Moderate vs. higher intensity (50% vs. 70%)
Paradis-Deschênes et al., 2020	RCT	IPC: 11, 31.5 ± 3.0 y; PLA: 9, 28.1 ± 2.5 y	Healthy	SIT + IPC (IPC)	4 wk; 2×/wk	PLA cuff
Pramkratok et al., 2022	RCT	RSH: 7, 24.14 ± 4.38 y; RSN: 7, 23.43 ± 4.86 y	Healthy	Repeated-sprint trg	6 wk; 3×/wk	NOR (RSN)
Prieur et al., 2013	Pre–post	1M, 19.7 ± 1.7 y	Healthy	Running HIIT	6 wk; 3×/wk	NA
De Smet et al., 2017	NRCT	18M, 23.9 ± 3.0 y	Healthy	Unilateral knee-extension HIIT (hypoxia vs. normoxia)	Hypoxia: 5 d/wk, ~15.5 h/d; HIIT: 3×/wk	HYP vs. NOR limb; no-trg CON
Chronic obstructive pulmonary disease (COPD) (n = 9)						
Barberan-Garcia et al., 2019	Pre–post	HS: 8M/2F, 65 ± 11 y; COPD: 15M/1F, 70 ± 5 y	COPD	Sup. interval cyc.	8 wk; 5 d/wk	NA
Borghi-Silva et al., 2008	RCT-X	16M	COPD	NA	NA	Sham ventilation (within-subject crossover CON)
Chiappa et al., 2008	CS	10M COPD pts (59.8 ± 7.9 y) and 11M sedentary controls (61.4 ± 6.3 y)	COPD	NA	NA	COPD vs. age- and sex-matched sedentary healthy CONs
Louvaris et al., 2017	CS	4M/2F; 67 ± 7	COPD	NA	NA	Within-subject: NOR (F_i_O_2_ 0.21) vs. hyperoxia (F_i_O_2_ 1.0)
de Paiva Azevedo et al., 2016	CS	13M COPD pts (64.3 ± 5.9 y) + 13M controls (63.0 ± 7.3 y)	COPD	NA	NA	COPD vs. age- and sex-matched sedentary healthy CONs
Prieur et al., 2019	RCT	5F/3M; 60.2 ± 6.7	COPD	NA	1 sess/cond.; 30 min constant-load cycling each; 30 min rest between sess	Crossover: FES-cyc. vs. PLA-FES
Puente-Maestu et al., 2003	Pre–post	21M, 63.0 ± 9.8 y	COPD (severe)	Endurance cyc.	6 wk; 3 d/wk	NA
Szucs et al., 2021	Pre–post	21M19F; 65.4 ± 7.4	COPD	Inpatient PR (4 wk)	4 wk; cyc./TM 2–3×/day, 20–30 min	NA
Vogiatzis et al., 2009	CS	13M COPD pts, 65 ± 2; 7M, healthy controls (60 ± 3)	COPD	NA	NA	COPD patients vs. age-matched healthy CONs
Peripheral artery disease (PAD)/intermittent claudication (n = 8)						
Baker et al., 2017	RCT	EX: 24M/5F, 66 y; CON: 17M/18F, 67 y	PAD	Sup. TM walk.	3 mo; 3×/wk	CON
Beckitt et al., 2012	NRCT	EX: 29M/13F, 66 ± 6.1 y; Angioplasty: 8M/4F, 68 ± 5.8 y	PAD	Sup. calf-focused circuit trg	12 wk	Ex. advice; angio. (revascularization) comparator
Cornelis et al., 2022	Pre–post	32M/9F, 70.3 ± 9.2 y	PAD	Hybrid walk prog.	12 wk; 2×/wk home + 1×/wk supervised	NA
Figoni et al., 2009	Pre–post	15M, 69 y	PAD (Fontaine IIa)	Combined sup. + home ex.	13 wk; 3×/wk clinic + 2×/wk home	NA
Li et al., 2021	RCT	EXP: 11M/3F, 66 y; CON: 6M/8F, 65 y	PAD	Sup. TM walk	3 mo; 3×/wk supervised	Usual care
Manfredini et al., 2020	RCT	13M, 69 ± 7 y	PAD	Revasc. vs. home walk	4 mo; 2×/day	Revascularization
Monteiro et al., 2019	RCT	CG: 14M/6F, 65.45 ± 10.60 y; MG: 14M/6F, 63.10 ± 10.54 y	PAD	Weighted walk	12 wk	Standard walk
Murrow et al., 2019	RCT	Oxygen-guided: 6M/2F, 72.0 ± 9.7 y; Pain-based: 8M/2F, 71.6 ± 8.8 y	PAD	NIRS-guided vs. pain-guided TM walk	12 wk; 3×/wk	Oxygen-guided vs. pain-guided
Chronic heart failure (CHF)/HFrEF (n = 5)						
Guimarães et al., 2021	NRCT	14M/10F: 47.5 ± 7.4 y	HFrEF	Heavy-load RT vs. heavy-load + BFR	12 wk; 3×/wk supervised	Usual care
Kravari et al., 2010	Pre–post	10M/4F; 45 ± 13 y	CHF	Aerobic cyc. ± RT	12 wk; 3×/wk	NA
Mezzani et al., 2013	RCT	CHF-AET: 15M, 65 ± 7 y; CHF-C: 15M, 63 ± 7 y; CON: 7M, 66 ± 4 y	CHF	Mod. aerobic trg	3 mo; 5×/wk	CHF-AET vs. CHF-C; healthy reference
Moalla et al., 2012	RCT	TG: 13.0 ± 1.4 y; CG 12.8 ± 1.3 y	CHF (NYHA II–III)	Home interval cyc.	12 wk; 3×/wk	No trg
Moreno et al., 2017	RCT	IMT: 8M/5F, 61 ± 14 y; CON: 8M/5F, 60 ± 13 y	CHF + IMW	Home IMT	8 wk; 6×/wk	CON
Congenital heart disease (n = 3)						
Lahti et al., 2022	Pre–post	CHD: 6M/4F, 13 ± 1 y; CTL: 4M/5F, 12 ± 3 y	Pediatric CHD	Home multi. ex.	12 wk; 3×/wk home + biwkly in-person	NA
Moalla et al., 2006	NRCT	18, 12–15 y	Pediatric CHD (repaired)	Home interval cyc.	12 wk; 3×/wk	No trg
Sandberg et al., 2019	CS	52M22F, 35.6 ± 14.3	ToF, Fontan/TCPC, dTGA Senning/Mustard, dTGA ASO, ccTGA, PA, and others with complex CHD	NA	NA	74 age- and sex-matched CONs
Pulmonary arterial hypertension (n = 1)						
Panagiotou et al., 2016	CS	25 pts	PAH	NA	NA	NA
Type 1/Type 2 diabetes (n = 3)						
Bauer et al., 2007	CS	T2DM (5M6F; 47 ± 4 y) and healthy controls (6M5F; 47 ± 6 y)	T2DM	NA	NA	11 age-, sex-, and BMI-matched healthy sedentary CONs
Molinari et al., 2013	Pre–post	FW (14M/6F; 66.0 ± 6.2 y), APA (10M/10F; 66.7 ± 5.7 y), healthy controls (8M/8F; 65.2 ± 3.9 y)	T2DM	Fit Walk. (FW) vs. APA (APA) APA	1 year; daily physical activity	16 age- and BMI-matched healthy CONs
Rissanen et al., 2018	NRCT	T1D: 8M, 33.4 ± 6.3 y; CON: 8M, 37.9 ± 7.1 y	T1DM	Unsup. individualized trg	1 yr; ~3–4 endurance + 1 resistance/wk	Lifestyle
Chronic kidney disease (CKD)/end-stage renal disease (n = 3)						
Theodorakopoulou et al., 2023	CS	Controls: 13M5F, 62.8 ± 11.4 y; CKD stage 2: 3M5F, 65.9 ± 9.9; stage 3a: 3M5F, 66.4 ± 10.4; stage 3b: 3M5F, 70.3 ± 9.9; stage 4: 3M5F, 67.3 ± 12.3	CKD	NA	NA	Healthy individuals or patients (each CKD stage group)
Wilkinson et al., 2019	CS	15M9F CKD pts, 58.3 ± 16.5 y; 1M5F, 47.2 ± 19.8	CKD (non-dialysis)	NA	NA	NA
Yao et al., 2024	CS	21M/4F; 57.6 ± 12.7 CKD, 15M/2F; 58.6 ± 7.0	ESRD/CKD; DM, HTN, CAD	NA	NA	NA
Other clinical populations (n = 4)						
Hiraoui et al., 2022	RCT	EXP: 20F, 49.71 ± 5.41 y; CON: 12F, 48.93 ± 4.76 y	Breast cancer (I–IIIA)	Multi. rehab	6 wk	Chemo only
Olivier et al., 2010	RCT	CG: 12M, 23.31 ± 3.12 y; TG: 12M, 25.11 ± 3.41 y	Post-ACLR	Rehab + one-leg cyc.	6 wk; 3×/wk	Contralateral leg
Porcelli et al., 2016	Pre–post	MM: 4M/2F, 51 ± 16 y; McA: 3M/4F, 41 ± 13 y	Metabolic myopathy	Home mod. cyc.	12 wk; 4 d/wk	NA
Van Hollebeke et al., 2022	RCT	INT: 13M/9F, 52 ± 18 y; CON: 9M/10F, 64 ± 7 y	ICU (MV, difficult wean)	High-intensity IMT	≤28 d; daily	Sham IMT

Notes: Studies are grouped by primary clinical subgroup, with alphabetical ordering preserved within each subgroup. Abbreviations: RCT, randomized controlled trial; NRCT, non-randomized controlled trial; RCT-X, randomized crossover trial; CS, cross-sectional/observational; M, male; F, female; y, years; wk, weeks; mo, months; sess, sessions; sup., supervised; cont., continuous; cyc., cycling; TM, treadmill; HIIT, high-intensity interval training; SIT, sprint interval training; RST, repeated-sprint training; BFR, blood flow restriction; IMT, inspiratory muscle training; RT, resistance training; FES, functional electrical stimulation; VHL, voluntary hypoventilation at low lung volume; HYP, hypoxia; NOR, normoxia; APA, adapted physical activity; PR, pulmonary rehabilitation; CON, control/no exercise; PLA, placebo; NA, not applicable; PAD, peripheral artery disease; COPD, chronic obstructive pulmonary disease; CHF, chronic heart failure; HFrEF, heart failure with reduced ejection fraction; CHD, congenital heart disease; PAH, pulmonary arterial hypertension; IMW, inspiratory muscle weakness; T1DM/T2DM, type 1/2 diabetes mellitus; CKD, chronic kidney disease; ESRD, end-stage renal disease; ACLR, anterior cruciate ligament reconstruction; ICU, intensive care unit; MV, mechanically ventilated; CAD, coronary artery disease; HTN, hypertensive.

**Table 2:** NIRS Methodology of Included Studies.

Study	NIRS Device	Muscle	S-D/Rate	NIRS Outcomes	Test Protocol
Incremental/ramp exercise (n = 19)					
Barberan-Garcia et al., 2019 [24]	InSpectra TS (325)	VM (L)	25 mm; 0.33 Hz	tHb; StO_2_	CWR cyc. (70% Wpeak)
Bauer et al., 2007 [52]	Optiplex TS	VL (dom)	2.0–3.5 cm; 50 Hz	HHb; τ/kin.	Incr. cyc. (10–20 W/min)
Borghi-Silva et al., 2008 [32]	NIRO 200	VL (L)	NS	HHb; O_2_Hb; tHb; TOI	Incr. cyc. (±PAV)
Breese et al., 2025 [34]	NIRO-200NX CW	VL (R)	3 cm; 10 Hz	TOI; Δ slopes	Incr. cyc. (+20 W/min) (+AO cal.)
Cornelis et al., 2022 [38]	PortaMon CW/SRS	Calf (affected)	30/35/40 mm; 10 Hz	TSI; AUC; rec. rate	Incr. cyc.
Espinosa-Ramírez et al., 2023 [53]	MOXY	IC (R)	NR; 2 Hz	HHb; O_2_Hb; tHb; TSI; rec. rate	Incr. cyc. (+20 W/2 min)
Guimarães et al., 2021 [25]	ISS OxiplexTS FD	VL (L)	3.0–4.4 cm; 100 Hz	O_2_Hb; HHb; tHb (rest, peak)	Incr. cyc. (5–10 W/min)
Kime et al., 2010 [64]	ASTEM multi-channel SRS-NIRS	VL (L)	20 & 30 mm; NR	StO_2_; SmO_2_; heterogeneity	Incr. cyc.
Lahti et al., 2022 [65]	NIRO-200NX CW	VL (R)	3 cm; NR	TOI	Peak VO_2_ cyc.: +25 W/2 min
Manfredini et al., 2020 [48]	Oxymon Mk III CW	GAS (medial calf)	3.5 cm; 1 Hz	AUC	Incr. cyc.
Mezzani et al., 2013 [68]	Omron HEO-100 CW	VL	NR; NR	HHb; O_2_Hb	Incr. cyc. (+AO cal.)
Moalla et al., 2006 [75]	NIM RunMan CW	SA	3 cm; NR	Resp. muscle oxygenation (%sat)	Incr. cyc. (10–20 W/min)
Paradis-Deschênes et al., 2020 [83]	PortaMon CW/SRS	VL (untreated)	40 mm; 10 Hz	ΔHHb; ΔtHb; ΔTSI	Incr. cyc.
Porcelli et al., 2016 [86]	PortaMon CW	VL	NR; NR	HHb; O_2_Hb; τ/kin.	Incr. cyc. (+AO cal.)
Prieur et al., 2013 [73]	NIMS 8-channel cwNIRS	VL (R)	3 cm; 3 Hz	HHb; tHb; heterogeneity	Incr. cyc. (+35 W/2 min)
Prieur et al., 2019 [84]	PortaMon	VL	30–40 mm; 1 Hz; DPF = 4	HHb; O_2_Hb; StO_2_; τ/kin.	Incr. cyc.
Rissanen et al., 2018 [57]	Oxymon Mk III CW	VL (R)	35–45 mm; 10 Hz	HHb	Incr. cyc.
Vogiatzis et al., 2009 [81]	InSpectra TS 325	VL	2.5 cm; 0.33 Hz	StO_2_; τ/kin.; Δ slopes	Incr. cyc. (5–10 W/min)
Wilkinson et al., 2019 [87]	BSXInsight CW	GAS-soleus (calf)	NS	SmO_2_	Incr. cyc.
Constant work rate cycling (n = 5)					
Chiappa et al., 2008 [37]	NIRO 200	VL (L)	NS	HHb; τ/kin.	CWR cyc. (75% peak)
Hamasaki et al., 2018 [54]	OmegaWave BOM-L1TRW	VL (R)	15 & 40 mm; 10 Hz	HHb; τ/kin.	CWR cyc. (80% VO_2_)
Kravari et al., 2010 [85]	InSpectra	Quadriceps	NR; NR	StO_2_; τ/kin.	CWR cyc.
Monteiro et al., 2019 [49]	PortaMon CW	GAS (med)	NR; 10 Hz	StO_2_; τ/kin.	CWR cyc. (+AO cal.)
Puente-Maestu et al., 2003 [77]	CWS 2000 RunMan	VL	NR; 0.83 Hz	O_2_Hb; τ/kin.	CWR cyc.
Step transitions (constant-load kinetics) (n = 4)					
Bailey et al., 2009 [31]	NIRO-300	VL (R)	NR; 2 Hz	HHb; O_2_Hb; tHb; τ/kin.	Step transitions (mod./severe)
McKay et al., 2009 [67]	NIRO-300	VL (quadriceps)	5 cm; NR	tHb; τ/kin.	Step transitions (mod./severe)
Murias et al., 2010 [50]	NIRO-300	VL	5 cm; 2 Hz	τ/kin.	Step transitions (mod./severe)
Murias et al., 2016 [56]	NIRO-300	VM (R)	5 cm; 2 Hz	HHb; τ/kin.	Step transitions (mod./severe)
Repeated-sprint/Wingate/running sprints (n = 8)					
Bourgeois et al., 2025 [33]	PortaMon CW/SRS	VL (R)	20 mm; 10 Hz	HHb; O_2_Hb	Wingate
Buchheit & Ufland, 2011 [35]	PortaMon CW/SRS	VL	NR; 10 Hz	TSI; rec. rate; Δ slopes	Repeated sprints
Caen et al., 2019 [36]	NIRO-200NX SRS	VL (R)	NR; 0.5 Hz	HHb; O_2_Hb; tHb; TOI; Δ slopes	Repeated sprints
De Smet et al., 2017 [39]	NIRO-200	VL	NR; 2 Hz	HHb; O_2_Hb; tHb; TOI	Repeated sprints
Faiss et al., 2015 [40]	PortaMon	TB (R)	40 mm; 20 Hz	HHb; tHb	Repeated double-poling sprints
Gatterer et al., 2018 [41]	NIRO-200	VL	NR; NR	tHb; TOI	Wingate
Lapointe et al., 2020 [66]	PortaMon CW/SRS	GAS (lat)	NR; 10 Hz	O_2_Hb	Repeated-sprint running
Pramkratok et al., 2022 [72]	PortaMon CW	VL (untreated)	NR; 10 Hz	ΔTSI; ΔO_2_Hb; ΔHHb; ΔtHb	Running anaerobic sprint test (6 × 35 m)
Resistance exercise/isometric tasks (n = 11)					
Bergamasco et al., 2025 [88]	Oxymon CW	VL	NR; 10 Hz	HHb; tHb; AUC	Resistance ex.
Costilla et al., 2023 [59]	NIRO-200NX	VM + VL	3 cm; NR	tHb; TOI	Squats (1RM + 8-rep)
Furuichi et al., 2009 [62]	NIRO-300	VL	4 cm; 1 kHz	HHb; O_2_Hb; τ/kin.	Isometric knee ext. (50% MVC)
Goto et al., 2019 [42]	ASTEM HB14-2 CW	TB (R)	30 mm; NR	AUC	Resistance ex.
Hiraoui et al., 2022 [43]	PortaMon	RF	NR; 10 Hz	ΔHHb during endurance test	Isometric endurance
Ida & Sasaki, 2024 [44]	ASTEM Oxy-Pro	BB (dom)	20 & 30 mm; 10 Hz	HHb; O_2_Hb; tHb; StO_2_	Low-load RT + BFR
Lin et al., 2023 [47]	ISS Imagent FD	VL	1.95–3.53 cm; 5 Hz	SpO_2_; τ/kin.	Isometric knee ext.
Moalla et al., 2012 [55]	NIM RunMan	VL	~3 cm; ~30 Hz	Deoxy/reoxy indices	Isometric knee ext. (50% MVC)
Molinari et al., 2013 [69]	NIRO-300	TA (L)	4 cm; 2 Hz	HHb; O_2_Hb; TOI; entropy; wavelet	Ankle flexo—ext.
Sandberg et al., 2019 [14]	InSpectra 325	Deltoid (dom)	25 mm; 0.29 Hz	HHb; StO_2_; rec. rate; Δ slopes	Resistance ex.
Theodorakopoulou et al., 2023 [79]	PortaMon	Forearm flexors (dom)	NS	tHb; TSI; rec. rate; Δ slopes	Resistance ex. (+AO cal.)
Treadmill walking protocols (n = 3)					
Baker et al., 2017 [23]	Custom DCS + FD-NIRS	GAS	2.2–3.8 cm (FD); 2.5 cm (DCS); 0.13 Hz	StO_2_; OEF; BFI	Gardner TM
Beckitt et al., 2012 [58]	NIRO-300 SRS	GAS (symp)	40 mm; 2 Hz	StO_2_; rec. rate	TM walk (2.5 km/h) (+AO cal.)
Figoni et al., 2009 [61]	InSpectra TS (325)	GAS/soleus (medial)	NR; 0.25–0.33 Hz	StO_2_; AUC	Gardner TM
Vascular occlusion/reactive hyperemia (n = 4)					
Horiuchi et al., 2023 [63]	OmegaWave BOM-L1TRW	GAS (med)	4 cm; NR	StO_2_; AUC	AO + reactive hyperemia
Jones et al., 2020 [45]	In-house broadband (mini-CYRIL)	GAS (lat)	NR; NR	HHb; O_2_Hb; oxCCO	AO + reactive hyperemia
Li et al., 2021 [46]	Custom FD-DOS (AO-DOS/VO-DOS)	Calf flexors (symp leg)	2.2–3.8 cm (FD); 2.5 cm (AO/VO-DOS); 0.13 Hz	tHb; StO_2_; OEF; mVO_2_	AO + reactive hyperemia
Murrow et al., 2019 [76]	PortaMon CW + Oxymon Mk III CW	Calf/GAS (affected)	2.5–5.5 cm; 1–10 Hz	O_2_Hb; mVO_2_	AO + reactive hyperemia
Inspiratory loading/respiratory muscle (n = 2)					
Moreno et al., 2017 [70]	ISS OxiplexTS	IC (L)	3.6–4.4 cm; NR	O_2_ saturation; deoxy-Hb (insp.)	Insp. loading to failure
Van Hollebeke et al., 2022 [80]	NIRO-200NX CW	SCL + SCM	40 mm; 5 Hz	StO_2_	Insp. loading to failure
Resting-state/provocative non-exercise (n = 3)					
Louvaris et al., 2017 [74]	3× NIRO-200	VL (R)	40 mm; NR	StO_2_; heterogeneity; Q	Resting (NOR vs. hyperoxia)
Szucs et al., 2021[78]	Moxy Monitor	VL	12.5–25 mm; 0.5 Hz	tHb; SmO_2_	Resting only
Yao et al., 2024 [82]	PortaMon	FDP (forearm)	30/35/40 mm; 10 Hz	O_2_Hb; tHb; TSI; wavelet	Resting only
Other protocols (NMES, one-leg cycling) (n = 3)					
de Paiva Azevedo et al., 2016 [60]	OxiplexTS	VL (R)	1.5–5.0 cm; 1 Hz	HHb; tHb; SmO_2_; OEF; τ/kin.	NMES protocol
Olivier et al., 2010 [51]	NIMS 8-channel cwNIRS	VL (untreated)	3 cm; 3 Hz	mVO_2_; LMBV	One-leg cyc.
Panagiotou et al., 2016 [71]	NIRO-200NX	VL (bilaterally)	NS	StO_2_	Multi-condition (rest, exercise)

Notes: CW, continuous wave; FD, frequency domain; SRS, spatially resolved spectroscopy; DCS, diffuse correlation spectroscopy; DOS, diffuse optical spectroscopy; S-D, source–detector spacing; VL, vastus lateralis; VM, vastus medialis; RF, rectus femoris; GAS, gastrocnemius; TA, tibialis anterior; TB, triceps brachii; BB, biceps brachii; IC, intercostals; SA, serratus anterior; SCL, scalene; SCM, sternocleidomastoid; FDP, flexor digitorum profundus; (R), right; (L), left; (med), medial; (lat), lateral; (symp), symptomatic; (dom), dominant; HHb, deoxygenated hemoglobin; O_2_Hb, oxygenated hemoglobin; tHb, total hemoglobin; StO_2_, tissue oxygen saturation; TSI, tissue saturation index; TOI, tissue oxygenation index; SmO_2_, muscle oxygen saturation; OEF, oxygen extraction fraction; BFI, blood flow index; mVO_2_, muscle oxygen consumption; AUC, area under curve; oxCCO, oxidized cytochrome c oxidase; LMBV, leg muscle blood volume; Q, regional blood flow; τ/kin., time constant/kinetics; rec., recovery; CWR, constant work rate; incr., incremental; cyc., cycling; TM, treadmill; ex., exercise; mod., moderate; AO, arterial occlusion; VO, venous occlusion; cal., calibration; PAV, proportional assist ventilation; NMES, neuromuscular electrical stimulation; MVC, maximal voluntary contraction; NOR, normoxia; NS, not stated; NR, not reported; Hz, Hertz; cm, centimeters; DPF, differential pathlength factor.

**Table 3:** Certainty of evidence for exercise modalities targeting muscle oxygenation.

Modality	*n*	Phenotype	Key NIRS Effects	Healthy/Trained	Clinical	Principal Limitations	Refs.
SIT/RST	10	OU-dom	↑ Deoxy amplitude; faster HHb kin.; faster reox	High (*n* = 9)	Very low (*n* = 1, T1D)	Skewed to healthy/trained; one null clinical study	[31,33,35,39,40,41,42,43,57,83]
HIIT	9	OU-dom	Faster HHb kin.; ↑ reox rate; correlates with VO_2_ kin.	High (*n* = 6)	Low (*n* = 3, PAD + COPD)	Heterogeneous protocols; few SIT comparisons	[24,34,36,38,49,51,53,63,67]
WBP (PAD)	8	MV/D-dom	↑ O_2_ extraction; capillarization; ABI unchanged	n/a	High (*n* = 8, PAD)	Population-specific; not generalizable	[23,46,47,48,52,58,61,86]
CAT	6	MV/D-dom	Restored Δ[HHb]/ΔVO_2_ matching; rapid onset	Moderate (*n* = 5)	Very low (*n* = 1)	Heterogeneity unchanged at 6 wk; age plateau	[50,56,59,64,67,84]
Combined/MMR	5	ML-imp	Gains in StO_2_ recovery, extraction, VO_2_ kin.	n/a	Moderate (*n* = 5, mixed)	Components unidentifiable; *n* ≤ 1–2 per population	[25,55,60,71,72]
RT	3	OU-dom (prop.)	Mixed; 40% VL failed to improve oxygenation	Low (*n* = 2)	Low (*n* = 1, CKD)	Non-linear dose–response; hypertrophy ≠ oxy gain	[44,45,87]
BFR	3	MV/D-dom (prop.)	Augments deoxy as add-on in healthy; impairs endurance at low load; one HFrEF study	Low (*n* = 5)	No data	Heterogeneous protocols and populations; not generalizable	[25,33,34,44,63]
IMT	3	CD-dom	Attenuated resp. metaboreflex; ↑ resp. muscle oxy	n/a	Very low (*n* = 2, 1 CHF outpatient, 1 ICU)	Heterogeneous clinical contexts; pooling not appropriate	[53,70,80]
Other	3	Varies	Varies; 15% desat threshold ≈ pain-guided in PAD	Very low (*n* = 1)	Very low (*n* = 2)	Single-study findings	[54,65,73]

Notes: Certainty rated per the four-criterion framework described in Section 3.7, with criteria mapped onto the imprecision/inconsistency, inconsistency, risk-of-bias, and indirectness/biological-gradient domains of GRADE. “n/a” indicates a modality not studied in the column’s population; “No data” indicates an evidence gap explicitly identified by this review. “↑” indicates a reported increase in the corresponding NIRS measure. ABI, ankle–brachial index; BFR, blood flow restriction; CAT, continuous aerobic training; CD-dom, convective delivery dominant; CHF, chronic heart failure; CKD, chronic kidney disease; COPD, chronic obstructive pulmonary disease; deoxy, deoxygenation; Δ[HHb]/ΔVO_2_, deoxygenation-to-VO_2_ ratio; HHb, deoxygenated hemoglobin; HIIT, high-intensity interval training; ICU, intensive care unit; IMT, inspiratory muscle training; kin., kinetics; MMR, multimodal rehabilitation; ML-imp, multi-level impairment; MV/D-dom, microvascular/diffusive dominant; *n*, number of studies; OU-dom, oxidative utilization dominant; oxy, oxygenation; PAD, peripheral artery disease; prop., proposed; reox, reoxygenation; resp., respiratory; RST, repeated-sprint training; RT, resistance training; SIT, sprint interval training; StO_2_, tissue oxygen saturation; T1D, type 1 diabetes; VL, velocity loss; VO_2_, oxygen uptake; WBP, walking-based program; wk, week.

**Table 4:** Proposed minimum reporting checklist for NIRS-based skeletal muscle oxygenation studies.

Domain	Item	Required Specification/Rationale
Instrumentation	Device name and manufacturer	Name, manufacturer, model, country.
	Technology type	CW, FD, BB, or time-resolved; determines absolute vs. relative measurement.
	Wavelengths used	All emission wavelengths (nm).
Probe configuration	Source–detector spacing	Measured as mm; list each distance if multiple. Determines penetration depth.
	Sampling rate	Measured as Hz. Required for kinetic resolution.
	Target muscle and probe placement	Muscle, anatomical landmarks, lateralization (dom/non-dom or affected/unaffected).
	Probe fixation and shielding	Attachment method and light shielding approach.
Tissue corrections	Adipose tissue thickness	Skinfold or ultrasound at probe site. Fat attenuates NIRS signal non-linearly.
	Differential pathlength factor (DPF)	DPF value with rationale (e.g., age- or sex-specific).
Calibration and analysis	Calibration approach	Baseline referencing, cuff occlusion (pressure, duration), manufacturer, or other.
	Signal processing	Filter cutoffs, motion–artifact handling, normalization.
	Analysis software and version	Software package and version.
Outcome reporting	Outcome variable nomenclature	Define each variable (StO_2_, TSI, TOI, SmO_2_) with device-specific terminology.
	Kinetic parameter derivation	If τ, TD, MRT, T50 reported: model, fitting window, goodness of fit.
	Reporting units	Measured as μM, arbitrary units, % of baseline, % of calibration range, etc.
Test protocol	Exercise or provocative test specification	Modality, intensity, duration, work–rest ratio for intervals.
	Test conditions	Body position, temperature, time of day, fasting, medication holds.
	Concurrent measurements	VO_2_, HR, EMG, lactate, BP, and their synchronization with NIRS.

Notes: Items are grounded in peer-reviewed standards most directly applicable to NIRS-based skeletal muscle oxygenation research: the Cores of Reproducibility in Physiology guidelines for NIRS [8] and the updated systematic review of muscle oximetry in sports science by Perrey et al. [98]. No ISO standard specifically governs NIRS reporting in exercise physiology; tissue-simulating phantom calibration approaches discussed by Perrey et al. [98] are recommended where feasible for cross-device comparability. The checklist is intended as a minimum dataset; additional items may be required for specific device platforms or measurement modalities.

## Data Availability

No new data were created or analyzed in this study. Data sharing is not applicable to this article.

## Supplementary Materials

The following supporting information can be downloaded at: https://www.mdpi.com/article/10.3390/diagnostics16111585/s1, Figure S1: Completeness of NIRS methodological reporting across included studies; Table S1: PEDro quality assessment of randomized controlled trials (n = 27); Table S2: Downs and Black quality assessment of non-randomized intervention studies (n = 24); Table S3: Newcastle–Ottawa Scale quality assessment of cross-sectional and observational studies (n = 10).

## Appendix A. Database-Specific Search Algorithms

The searches detailed below were executed prospectively in accordance with the registered protocol (OSF DOI: 10.17605/OSF.IO/QW8R3; PROSPERO CRD420261365040). Boolean search strategies were adapted from the canonical PubMed strategy (Appendix A.1) for each platform listed below. All searches were conducted from database inception through to April 2026 and restricted to English-language records. Animal-only studies and non-primary article types (reviews, editorials, comments, letters, case reports) were excluded at the database level when possible.

### Appendix A.1. PubMed (National Library of Medicine)

**Line**	**Search String**
#1	“Oxygen Consumption”[Mesh]
#2	“Spectroscopy, Near-Infrared”[Mesh]
#3	(“muscle oxygenation”[tiab] OR “tissue oxygenation”[tiab] OR “muscle oxygen saturation”[tiab] OR “tissue oxygen saturation”[tiab] OR “muscle deoxygenation”[tiab] OR “tissue hemoglobin”[tiab])
#4	(SmO_2_[tiab] OR StO_2_[tiab] OR StiO_2_[tiab] OR TSI[tiab] OR TOI[tiab] OR HHb[tiab] OR “deoxygenated hemoglobin”[tiab] OR O_2_Hb[tiab] OR “oxygenated hemoglobin”[tiab] OR tHb[tiab] OR “total hemoglobin”[tiab])
#5	#1 OR #3 OR #4
#6	(“near-infrared spectroscopy”[tiab] OR “near infrared spectroscopy”[tiab] OR NIRS[tiab] OR “muscle oximetry”[tiab] OR “tissue oximetry”[tiab])
#7	#2 OR #6
#8	“Exercise”[Mesh] OR “Exercise Therapy”[Mesh] OR “High-Intensity Interval Training”[Mesh] OR “Resistance Training”[Mesh] OR “Rehabilitation”[Mesh]
#9	(exercise [tiab] OR training[tiab] OR “aerobic training”[tiab] OR “endurance training”[tiab] OR “resistance training”[tiab] OR “strength training”[tiab] OR HIIT[tiab] OR “high-intensity interval training”[tiab] OR “sprint interval training”[tiab] OR SIT[tiab] OR “repeated sprint”[tiab] OR “continuous training”[tiab] OR “blood flow restriction”[tiab] OR BFR[tiab] OR “inspiratory muscle training”[tiab] OR IMT[tiab] OR rehabilitation[tiab] OR “pulmonary rehabilitation”[tiab] OR “cardiac rehabilitation”[tiab] OR “walking training”[tiab] OR “treadmill training”[tiab] OR cycling[tiab])
#10	#8 OR #9
#11	#5 AND #7 AND #10
#12	#11 AND English[lang]
#13	#12 NOT ((“Animals”[Mesh] NOT “Humans”[Mesh]) OR “Case Reports”[pt] OR “Editorial”[pt] OR “Review”[pt] OR “Comment”[pt])
Records identified: 1519.	

### Appendix A.2. Web of Science Core Collection (Clarivate)

**Line**	**Search String**
#1	TS = (“muscle oxygenation” OR “tissue oxygenation” OR “muscle oxygen saturation” OR “tissue oxygen saturation” OR “muscle deoxygenation”)
#2	TS = (SmO_2_ OR StO_2_ OR StiO_2_ OR TSI OR TOI OR HHb OR “deoxygenated hemoglobin” OR O_2_Hb OR “oxygenated hemoglobin” OR tHb OR “total hemoglobin”)
#3	#1 OR #2
#4	TS = (“near-infrared spectroscopy” OR “near infrared spectroscopy” OR NIRS OR “muscle oximetry” OR “tissue oximetry”)
#5	TS = (exercise* OR training OR “aerobic training” OR “endurance training” OR “resistance training” OR “strength training” OR HIIT OR “high-intensity interval training” OR “sprint interval training” OR SIT OR “repeated sprint” OR “continuous training” OR “blood flow restriction” OR BFR OR “inspiratory muscle training” OR IMT OR rehabilitation OR “pulmonary rehabilitation” OR “cardiac rehabilitation” OR “walking training” OR “treadmill training” OR cycling)
#6	#3 AND #4 AND #5
#7	#6, refined by: Languages = ENGLISH; Document Types = ARTICLE (excluding REVIEW, EDITORIAL MATERIAL, MEETING ABSTRACT, LETTER)
Records identified: 1110.	

### Appendix A.3. Scopus (Elsevier)

**Line**	**Search String**
#1	TITLE-ABS-KEY(“muscle oxygenation” OR “tissue oxygenation” OR “muscle oxygen saturation” OR “tissue oxygen saturation” OR “muscle deoxygenation”)
#2	TITLE-ABS-KEY(SmO_2_ OR StO_2_ OR StiO_2_ OR TSI OR TOI OR HHb OR “deoxygenated hemoglobin” OR O_2_Hb OR “oxygenated hemoglobin” OR tHb OR “total hemoglobin”)
#3	#1 OR #2
#4	TITLE-ABS-KEY(“near-infrared spectroscopy” OR “near infrared spectroscopy” OR NIRS OR “muscle oximetry” OR “tissue oximetry”)
#5	TITLE-ABS-KEY(exercise OR training OR “aerobic training” OR “endurance training” OR “resistance training” OR “strength training” OR HIIT OR “high-intensity interval training” OR “sprint interval training” OR SIT OR “repeated sprint” OR “continuous training” OR “blood flow restriction” OR BFR OR “inspiratory muscle training” OR IMT OR rehabilitation OR “pulmonary rehabilitation” OR “cardiac rehabilitation” OR “walking training” OR “treadmill training” OR cycling)
#6	#3 AND #4 AND #5
#7	#6 AND (LIMIT-TO(LANGUAGE, “English”)) AND (LIMIT-TO(DOCTYPE, “ar”)) AND (EXCLUDE(DOCTYPE, “re”) AND EXCLUDE(DOCTYPE, “ed”) AND EXCLUDE(DOCTYPE, “le”) AND EXCLUDE(DOCTYPE, “no”) AND EXCLUDE(DOCTYPE, “cp”))
Records identified: 922.	

### Appendix A.4. Cochrane Central Register of Controlled Trials (CENTRAL)

**Line**	**Search String**
#1	[mh “Spectroscopy, Near-Infrared”]
#2	(“near-infrared spectroscopy” OR “near infrared spectroscopy” OR NIRS OR “muscle oximetry” OR “tissue oximetry”):ti, ab, kw
#3	#1 OR #2
#4	(“muscle oxygenation” OR “tissue oxygenation” OR “muscle oxygen saturation” OR “tissue oxygen saturation” OR “muscle deoxygenation”):ti, ab, kw
#5	(SmO_2_ OR StO_2_ OR StiO_2_ OR TSI OR TOI OR HHb OR “deoxygenated hemoglobin” OR O_2_Hb OR “oxygenated hemoglobin” OR tHb OR “total hemoglobin”):ti, ab, kw
#6	#4 OR #5
#7	[mh Exercise] OR [mh “Exercise Therapy”] OR [mh “High-Intensity Interval Training”] OR [mh “Resistance Training”] OR [mh Rehabilitation]
#8	(exercise OR training OR “aerobic training” OR “endurance training” OR “resistance training” OR “strength training” OR HIIT OR “high-intensity interval training” OR “sprint interval training” OR SIT OR “repeated sprint” OR “continuous training” OR “blood flow restriction” OR BFR OR “inspiratory muscle training” OR IMT OR rehabilitation OR “pulmonary rehabilitation” OR “cardiac rehabilitation” OR “walking training” OR “treadmill training” OR cycling):ti, ab, kw
#9	#7 OR #8
#10	#3 AND #6 AND #9 in Trials
Records identified: 335.	

### Appendix A.5. EMBASE (Elsevier, Embase.com Platform)

Free-text terms were applied to title, abstract, and author keyword fields (:ti, ab, kw). Emtree subject headings were exploded (/exp) to include narrower terms. The [medline]/lim filter was applied to exclude records originating from MEDLINE, avoiding duplication with the PubMed search (Appendix A.1).

**Line**	**Search String**
#1	‘oxygen consumption’/exp
#2	‘near infrared spectroscopy’/exp
#3	(‘muscle oxygenation’ OR ‘tissue oxygenation’ OR ‘muscle oxygen saturation’ OR ‘tissue oxygen saturation’ OR ‘muscle deoxygenation’ OR ‘tissue hemoglobin’):ti, ab, kw
#4	(SmO_2_ OR StO_2_ OR StiO_2_ OR TSI OR TOI OR HHb OR ‘deoxygenated hemoglobin’ OR O_2_Hb OR ‘oxygenated hemoglobin’ OR tHb OR ‘total hemoglobin’):ti, ab, kw
#5	#1 OR #3 OR #4
#6	(‘near-infrared spectroscopy’ OR ‘near infrared spectroscopy’ OR NIRS OR ‘muscle oximetry’ OR ‘tissue oximetry’):ti, ab, kw
#7	#2 OR #6
#8	‘exercise’/exp OR ‘kinesiotherapy’/exp OR ‘high intensity interval training’/exp OR ‘resistance training’/exp OR ‘rehabilitation’/exp
#9	(exercise OR training OR ‘aerobic training’ OR ‘endurance training’ OR ‘resistance training’ OR ‘strength training’ OR HIIT OR ‘high-intensity interval training’ OR ‘sprint interval training’ OR SIT OR ‘repeated sprint’ OR ‘continuous training’ OR ‘blood flow restriction’ OR BFR OR ‘inspiratory muscle training’ OR IMT OR rehabilitation OR ‘pulmonary rehabilitation’ OR ‘cardiac rehabilitation’ OR ‘walking training’ OR ‘treadmill training’ OR cycling):ti, ab, kw
#10	#8 OR #9
#11	#5 AND #7 AND #10
#12	#11 AND [english]/lim
#13	#12 NOT (‘animal’/exp NOT ‘human’/exp)
#14	#13 NOT (‘case report’/de OR ‘review’/it OR ‘editorial’/it OR ‘note’/it OR ‘letter’/it OR ‘conference abstract’/it)
#15	#14 NOT [medline]/lim
Records identified: 613.	

### Appendix A.6. Physiotherapy Evidence Database (PEDro)

PEDro provides a simple-search interface only and does not support Boolean line-numbered search strategies. The Advanced Search interface was used with the following field entries.

**Field**	**Entry**
Abstract & Title	near-infrared spectroscopy
Therapy	fitness training
Body Part	lower leg or knee, or upper leg or hip
Method	clinical trial
Published Since	(no restriction)

Supplementary searches were also conducted in the abstract and title field using the terms “NIRS,” “muscle oxygenation,” and “tissue oxygenation” individually, followed by manual screening. PEDro searches return records as a single result list without Boolean combinations; each term was searched separately, and results were merged and deduplicated externally in the reference management software.

Records identified: 14.

### Appendix A.7. ClinicalTrials.gov

ClinicalTrials.gov was searched using the Advanced Search interface (v2). Boolean strings were entered with field-specific tagging for condition, intervention, and outcome measures as shown below. Searches were restricted to interventional studies with results posted and with no date restriction.

**Line**	**Search String**
#1	AREA[ConditionSearch] (“peripheral artery disease” OR “COPD” OR “heart failure” OR “congenital heart disease” OR “diabetes” OR “chronic kidney disease” OR “critical illness” OR healthy)
#2	AREA[InterventionSearch] (exercise OR training OR rehabilitation OR HIIT OR “sprint interval training” OR “resistance training” OR “blood flow restriction” OR “inspiratory muscle training”)
#3	AREA[OutcomeMeasure] (“near-infrared spectroscopy” OR NIRS OR “muscle oxygenation” OR “tissue oxygenation” OR “muscle oxygen saturation” OR StO_2_ OR SmO_2_ OR “muscle deoxygenation”)
#4	#1 AND #2 AND #3
#5	#4 filtered by Study Type = Interventional; Study Status = with results

Trial records identified through ClinicalTrials.gov were screened for published peer-reviewed reports of completed primary outcomes; conference abstracts and unpublished trial postings were excluded from full-text eligibility assessment, consistent with the inclusion criteria specified in Section 2.3.

Records identified: 440.

Search dates, platform versions, and yield by database.

**Database**	**Platform/Version**	**Date Range**	**Records Identified**
PubMed	PubMed via NCBI	Inception to April 2026	1519
Web of Science	Web of Science Core Collection (Clarivate)	Inception to April 2026	1110
Scopus	Scopus (Elsevier)	Inception to April 2026	922
EMBASE	Embase.com (Elsevier)	Inception to April 2026	613
ClinicalTrials.gov	NLM (Advanced Search v2)	Inception to April 2026	440
Cochrane CENTRAL	Cochrane Library, Wiley	Inception to April 2026	335
PEDro	Physiotherapy Evidence Database	Inception to April 2026	14
Total			4953

## Appendix B. Standardized Data Extraction Form

This standardized form was used by two independent reviewers (JL and TB) to extract data from each included study. The form captures four domains corresponding to those specified in Methods Section 2.5: (1) study identification, (2) participant characteristics, (3) intervention details, and (4) NIRS methodology. For each field, reviewers entered either the value reported in the original study, “NR” if the field was not reported, or “NA” if the field was not applicable to the study design. Discrepancies between independent extractions were identified field by field and resolved through consensus, with the corresponding author (YKJ) consulted when consensus could not be reached.

Italicized text in parentheses below each field provides extraction guidance and is not part of the extracted value.

Study record.

**Field**	**Extracted Value (or NR/NA)**
Reviewer name	(JL/TB)
Extraction date	(DD/MM/YYYY)
Study reference number	(as numbered in the bibliography)
First author and year	(e.g., Bailey et al. 2009)
DOI or PubMed ID	(for tracking)

Domain 1. Study identification.

**Field**	**Extracted Value (or NR/NA)**
Authors	(all authors as listed)
Year of publication	(YYYY)
Journal	(full journal name)
Country of corresponding author	(country only)
Funding source	(as declared by authors; NR if not reported)
Conflict of interest declaration	(yes/no/NR)
Study design	(RCT/RCT-X/NRCT/pre–post/CS)
Registration	(registry name and ID; NR if absent)
Ethics approval	(IRB/committee name and reference number; NR if absent)

Domain 2. Participant characteristics.

**Field**	**Extracted Value (or NR/NA)**
Total sample size enrolled	(n)
Total sample size analyzed	(n; note dropouts if applicable)
Number of groups	(intervention groups, including control)
Sample size per group	(e.g., EX: 24; CON: 19)
Sex distribution per group	(n male/n female per group)
Age per group	(mean (SD) or median (IQR); years)
Body mass index per group	(kg/m^2^; NR if not reported)
Health status/population category	(healthy/clinical (specify condition))
Clinical diagnosis criteria	(if clinical; e.g., GOLD stage, NYHA class, ABI cutoff)
Disease severity/stage	(as reported)
Training status	(untrained/recreationally active/trained/athletic/NR)
Inclusion criteria	(key inclusion criteria as stated)
Exclusion criteria	(key exclusion criteria as stated)
Medications	(relevant medications; NR if not reported)
Comorbidities	(relevant comorbidities; NR if not reported)

Domain 3. Intervention details.

Complete this domain for intervention studies only; mark NA for cross-sectional/observational designs.

**Field**	**Extracted Value (or NR/NA)**
Exercise modality	(SIT/RST/HIIT/WBP/CAT/MMR/RT/BFR/IMT/Other)
Specific protocol description	(free text)
Exercise mode	(cycling/treadmill/running/resistance/other)
Total intervention duration	(weeks)
Session frequency	(sessions per week)
Sessions per week (supervised vs. unsupervised)	(if mixed)
Session duration	(minutes per session)
Intensity prescription	(e.g., 70% VO_2_max, RPE 15, %1RM, watts)
Sets/repetitions per session	(for resistance/sprint protocols)
Work—rest ratio	(for interval/sprint protocols)
Progression rule	(how intensity/volume progressed)
Comparator group(s)	(no exercise/sham/alternative training/usual care/other)
Adherence rate	(% of prescribed sessions completed)
Adverse events	(n events and description; NR if not reported)

Domain 4. NIRS methodology and outcomes
Instrumentation.

**Field**	**Extracted Value (or NR/NA)**
Device manufacturer	(e.g., Artinis, Moxy, Hamamatsu)
Device model	(e.g., PortaMon, PortaLite, NIRO-200NX)
NIRS technology	(continuous wave (CW)/frequency domain (FD)/broadband (BB)/spatially resolved spectroscopy (SRS)/diffuse correlation spectroscopy (DCS))
Number of wavelengths	(e.g., 2, 4, broadband)
Specific wavelengths used	(nm values; NR if not reported)

Probe placement and measurement.

**Field**	**Extracted Value (or NR/NA)**
Target muscle(s)	(e.g., vastus lateralis, gastrocnemius, biceps brachii)
Probe location relative to anatomical landmark	(e.g., 15 cm proximal to patella)
Probe side	(dominant/non-dominant/both/NR)
Source–detector spacing	(mm; if multiple, list all)
Sampling rate	(Hz)
Probe attachment method	(double-sided tape/bandage/custom holder; light shielding)
Skinfold thickness measurement	(yes (value and method)/no)
Adipose tissue thickness correction applied	(yes (method)/no)

Signal processing and calibration.

**Field**	**Extracted Value (or NR/NA)**
Calibration approach	(baseline referencing/cuff occlusion/manufacturer/other)
Normalization method	(physiological calibration/arbitrary units/delta from baseline/% of resting/other)
Differential pathlength factor (DPF) value	(if applicable; or NA)
Filtering/smoothing applied	(low-pass cutoff, moving average window, etc.)
Motion artifact handling	(method used; NR if not reported)

NIRS-derived outcome variables.

**Field**	**Extracted Value (or NR/NA)**
Primary NIRS outcome(s)	(e.g., [HHb], StO_2_, TSI, delta[HHb] amplitude)
Secondary NIRS outcome(s)	(as reported)
Kinetic parameters reported	(time constant (tau), time delay (TD), mean response time (MRT); list if reported)
Reoxygenation outcomes	(T50, half-recovery time, slope; list if reported)
Delta[HHb]/deltaVO_2_ ratio reported	(yes/no)
Units of reporting	(absolute (uM)/arbitrary/% of baseline/other)

Test protocol.

**Field**	**Extracted Value (or NR/NA)**
Testing modality	(incremental cycling/treadmill/arterial occlusion/isometric/sport-specific/other)
Test intensity	(max effort/submax/occlusion pressure (mmHg))
Test duration	(minutes or duration of each phase)
Pre-test rest	(supine/seated; duration)
Recovery monitoring duration	(post-exercise/post-occlusion; minutes)
Timing of NIRS assessment	(baseline/pre/immediate post/24 h/48 h/chronic)
Concurrent measurements	(e.g., VO_2_, heart rate, EMG, lactate)

Quality appraisal.

The appropriate tool was applied based on study design (see Section 2.6).

**Field**	**Extracted Value (or NR/NA)**
Quality appraisal tool used	(PEDro/Downs and Black/Newcastle–Ottawa Scale)
Total score	(as appropriate to the tool)
Items rated as not met (key items)	(list item numbers)
Reviewer comments on quality	(free text)

Extraction notes.

Use this section to document any ambiguities, judgment calls, or correspondence with study authors.
