# Supplementary material for: Near-Infrared Spectroscopy in the Pathophysiology, Diagnosis, and Exercise-Based Management of Muscle Oxygenation Impairment
Source: Diagnostics (Basel). 2026 May 22;16(11):1585. doi: 10.3390/diagnostics16111585 (PMC13256831; doi:10.3390/diagnostics16111585)
Supplement: Supplementary file 1 [file diagnostics-16-01585-s001.zip › diagnostics-4299352-supplementary.pdf]

Supplementary Materials

Contents

- Supplementary Table S1 — PEDro quality assessment (RCTs)
- Supplementary Table S2 — Downs and Black quality assessment (non-randomized studies)
- Supplementary Table S3 — Newcastle-Ottawa Scale quality assessment (observational studies)
- Supplementary Figure S1 — Completeness of NIRS methodological reporting

Supplementary Table S1. PEDro Quality Assessment of Randomized Controlled Trials (n = 27)

Study	C1	C2	C3	C4	C5	C6	C7	C8	C9	C10	C11	Total
Bailey et al., 2009	1	1	0	1	0	0	0	1	1	1	1	6
Baker et al., 2017	1	1	0	0	0	0	0	0	0	1	1	3
Borghi-Silva et al., 2008	1	1	0	1	1	1	0	1	1	1	1	8
Bourgeois et al., 2025	1	1	0	1	0	0	0	1	1	1	1	6
Breese et al., 2025	1	0	0	1	0	0	0	1	1	1	1	5
Costilla et al., 2023	1	1	0	0	0	0	0	1	0	1	1	4
Espinosa-Ramirez et al., 2023	1	1	0	0	0	0	0	1	0	1	1	4
Faiss et al., 2015	1	1	0	1	1	1	0	1	1	1	1	8
Furuichi et al., 2009	1	1	0	0	0	0	0	0	0	1	1	3
Gatterer et al., 2018	1	1	0	0	0	0	0	0	0	1	1	3
Goto et al., 2019	1	1	0	1	0	0	0	1	1	1	1	6
Hiraoui et al., 2022	1	1	1	1	0	0	0	0	0	1	1	5
Horiuchi et al., 2023	1	1	0	1	0	0	0	1	1	1	1	6
Lapointe et al., 2020	1	1	0	1	0	0	0	0	0	1	1	4
Li et al., 2021	1	1	0	1	0	0	0	1	1	1	1	6
Manfredini et al., 2020	1	1	0	1	0	0	0	0	0	1	1	4
McKay, Paterson & Kowalchuk, 2009	1	1	0	1	0	0	0	1	1	1	1	6
Mezzani et al., 2013	1	1	0	1	0	0	1	1	0	1	1	6
Moalla et al., 2012	1	1	0	1	0	0	0	1	1	1	1	6
Monteiro et al., 2019	1	1	0	1	0	0	1	0	0	1	1	5
Moreno et al., 2017	1	1	0	1	0	0	0	1	1	1	1	6
Murrow et al., 2019	1	1	0	1	0	0	0	0	0	1	1	4
Olivier et al., 2010	1	1	0	1	0	0	1	1	1	1	1	7
Paradis-Deschenes et al., 2020	1	1	0	1	1	0	0	0	0	1	1	5
Pramkratok et al., 2022	1	1	0	1	1	0	0	1	1	1	1	7
Prieur et al., 2019	1	0	0	0	0	0	0	1	1	0	1	3
Van Hollebeke et al., 2022	1	1	1	0	1	0	1	1	1	1	1	8

Notes: C1, eligibility criteria specified (not included in total score); C2, random allocation; C3, concealed allocation; C4, baseline comparability; C5, blinding of subjects; C6, blinding of therapists; C7, blinding of assessors; C8, >85% follow-up; C9, intention-to-treat analysis; C10, between-group statistical comparison; C11, point estimates and variability. Total score range: 0–10 (sum of C2–C11). 1 = criterion met; 0 = criterion not met or not reported.

Supplementary Table S2. Downs and Black Quality Assessment of Non-Randomized Intervention Studies (n = 24)

Study	1	2	3	4	5	6	7	8	9	10	11	12	13	14	15	16	17	18	19	20	21	22	23	24	25	26	27	Total	Quality
Barberan-Garcia et al., 2019	1	1	1	1	1	1	1	0	0	1	1	1	1	0	0	1	1	1	1	1	1	1	0	0	0	0	0	18	Fair
Beckitt et al., 2012	1	1	1	1	1	1	1	0	0	1	1	1	1	0	0	1	1	1	1	1	1	1	0	0	0	0	0	18	Fair
Buchheit & Ufland, 2011	1	1	1	1	1	1	1	0	0	1	1	1	1	0	0	1	1	1	1	1	1	1	0	0	0	0	0	18	Fair
Caen et al., 2019	1	1	1	1	1	1	1	0	0	1	1	1	1	0	0	1	1	1	1	1	1	1	0	0	0	1	1	20	Good
Cornelis et al., 2022	1	1	1	1	1	1	1	0	1	1	1	1	1	0	1	1	1	1	1	1	1	0	0	0	0	1	0	20	Good
De Smet et al., 2017	1	1	1	1	2	1	1	0	0	1	1	1	1	0	0	1	1	1	1	1	1	1	1	1	1	0	0	22	Good
Figoni et al., 2009	1	1	1	1	1	1	1	0	0	1	1	0	1	0	0	1	1	1	1	1	1	1	0	0	0	0	0	17	Fair
Guimaraes et al., 2021	1	1	1	1	2	1	1	1	1	1	1	1	1	0	0	1	1	1	1	1	1	1	1	0	1	1	0	24	Good
Hamasaki et al., 2018	1	1	1	1	1	1	1	0	0	1	1	1	1	0	0	1	1	1	1	1	1	1	0	0	0	0	0	18	Fair
Ida & Sasaki, 2024	1	1	1	1	1	1	1	0	0	1	1	1	1	0	0	1	1	1	1	1	1	1	0	0	0	0	1	19	Fair
Jones et al., 2020	1	1	1	1	0	1	1	0	0	1	1	1	1	0	0	1	1	1	0	1	1	1	0	0	0	0	0	16	Fair
Kime et al., 2010	1	1	1	1	0	1	1	0	0	1	1	1	1	0	0	1	1	1	0	1	1	1	0	0	0	0	0	16	Fair
Kravari	1	1	1	1	1	1	1	0	0	1	1	1	1	0	0	1	1	1	1	1	1	1	0	0	0	0	0	18	Fair

et al., 2010																													
Lahti et al., 2022	1	1	1	1	1	1	1	0	1	1	1	1	1	0	0	1	1	1	1	1	1	0	0	0	0	1	0	19	Fair
Lin et al., 2023	1	1	1	1	1	1	1	0	0	1	1	1	1	0	0	1	1	1	1	1	1	1	0	0	0	0	0	18	Fair
Moalla et al., 2006	1	1	1	1	2	1	1	1	0	1	1	1	1	0	0	1	1	1	1	1	1	1	0	0	1	0	0	21	Good
Molinari et al., 2013	1	1	1	1	1	1	1	0	0	1	1	1	1	0	0	1	1	1	0	1	0	1	0	0	0	0	0	16	Fair
Murias et al., 2010	1	1	1	1	1	1	1	0	0	1	1	1	1	0	0	1	1	1	1	1	1	1	0	0	0	0	0	18	Fair
Murias et al., 2016	1	1	1	1	1	1	1	0	0	1	1	1	1	0	0	1	1	1	0	1	1	1	1	0	0	0	0	18	Fair
Porcelli et al., 2016	1	1	1	1	1	1	1	0	0	1	1	1	1	0	0	1	1	1	1	1	1	1	0	0	0	0	0	18	Fair
Prieur & Mucci, 2013	1	1	1	1	0	1	1	0	0	1	1	1	1	0	0	1	1	1	0	1	1	1	0	0	0	0	0	16	Fair
Puente-Maestu et al., 2003	1	1	1	1	0	1	1	0	0	1	1	1	1	0	0	1	1	1	1	1	1	1	0	0	0	0	0	17	Fair
Rissanen et al., 2018	1	1	1	1	2	1	1	0	1	1	1	1	1	0	0	1	1	1	1	1	1	1	0	0	1	1	1	23	Good
Szucs et al., 2021	1	1	1	1	1	1	1	0	0	1	1	1	1	0	0	1	1	1	1	1	1	1	0	0	0	0	1	19	Fair

Notes: Items 1–10, Reporting; 11–13, External validity; 14–20, Internal validity – Bias; 21–26, Internal validity – Confounding; 27, Power. Total score range: 0–28. Quality levels: Excellent (≥26), Good (20–25), Fair (14–19), Poor (<14). 1 = criterion met; 0 = criterion not met or not reported.

Supplementary Table S3. Newcastle-Ottawa Scale Quality Assessment of Cross-Sectional and Observational Studies (n = 10)

Study	S1	S2	S3	S4	C1a	C1b	O1	O2	Total	Quality
Bauer et al., 2007	0	0	0	2	1	1	1	1	6	Satisfactory
Chiappa et al., 2008	1	0	0	2	1	1	1	1	7	Satisfactory
de Paiva Azevedo et al., 2016	1	1	0	2	1	1	1	1	8	Good
Louvaris et al., 2017	0	0	0	2	1	1	1	1	6	Satisfactory
Panagiotou et al., 2016	1	0	0	2	0	0	1	1	5	Satisfactory
Sandberg et al., 2019	1	0	1	2	1	1	1	1	8	Good
Theodorakopoulou et al., 2023	1	0	0	2	1	1	1	1	7	Satisfactory
Vogiatzis et al., 2009	0	0	0	2	1	1	1	1	6	Satisfactory
Wilkinson et al., 2019	1	0	0	2	1	1	1	1	7	Satisfactory
Yao et al., 2024	0	0	0	2	1	0	1	1	5	Satisfactory

Notes: S1, representativeness of sample; S2, sample size justification; S3, non-respondents; S4, ascertainment of exposure (max 2★ for validated tool); C1a, controls for most important confounder; C1b, controls for additional confounder; O1, assessment of outcome (max 2★ for independent blind assessment); O2, appropriateness of statistical test. Total stars range: 0–10. Quality levels: Good (≥8), Satisfactory (5–7), Unsatisfactory (<5).

Supplementary Figure S1. Completeness of NIRS methodological reporting across included studies

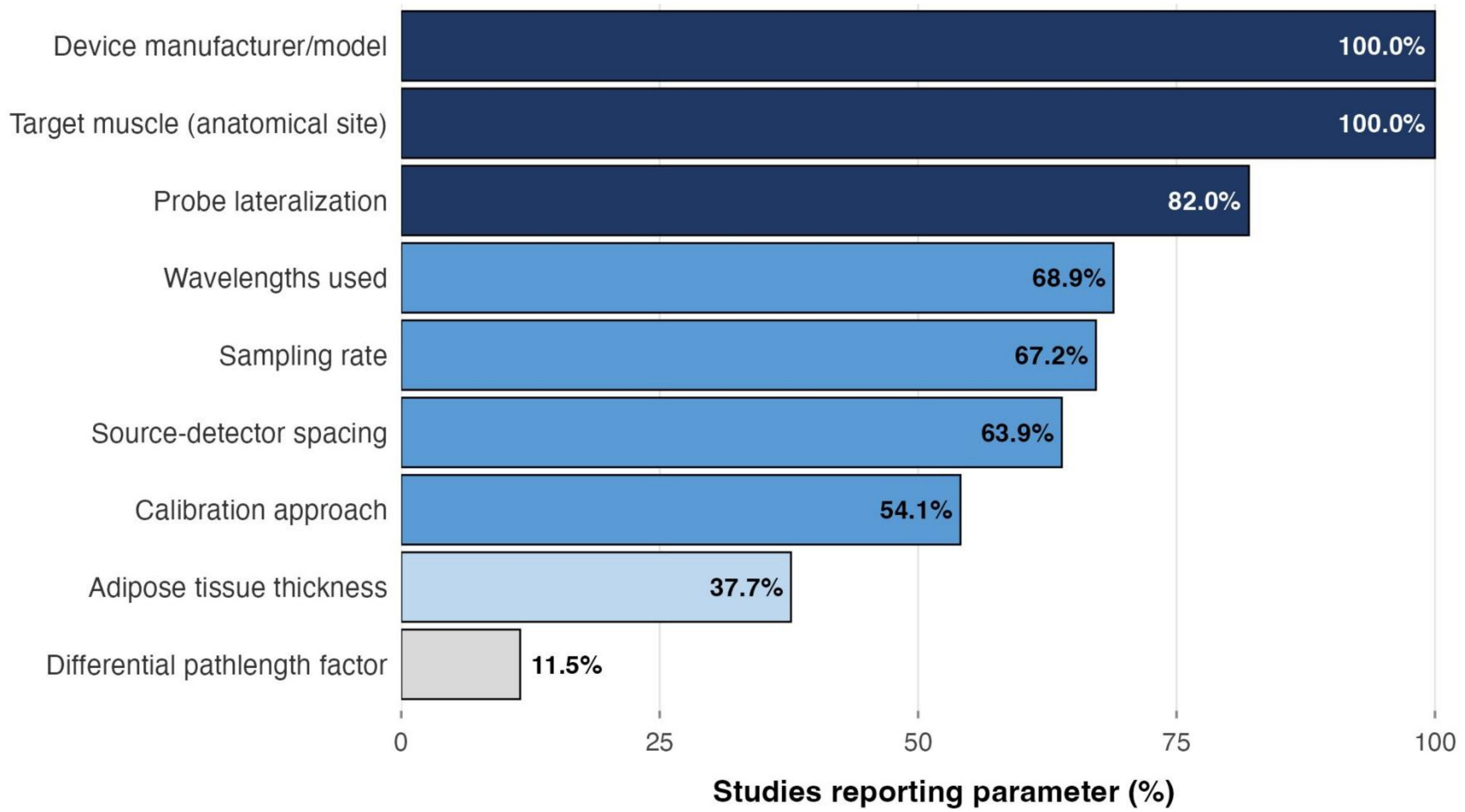

Figure S1. Percentage of included studies reporting each near-infrared spectroscopy (NIRS) methodological parameter. Bars are shaded by reporting completeness, from consistently reported parameters (device manufacturer/model and target muscle, both 100%) to infrequently reported parameters (differential pathlength factor, 11.5%). Parameters such as adipose tissue thickness (37.7%) and differential pathlength factor are critical determinants of NIRS signal validity yet remain underreported across the literature.

Disclaimer/Publisher's Note: The statements, opinions and data contained in all publications are solely those of the individual author(s) and contributor(s) and not of MDPI and/or the editor(s). MDPI and/or the editor(s) disclaim responsibility for any injury to people or property resulting from any ideas, methods, instructions or products referred to in the content.
